# Interpretable machine learning comprehensive human gait deterioration analysis

**DOI:** 10.3389/fninf.2024.1451529

**Published:** 2024-08-23

**Authors:** Abdullah S. Alharthi

**Affiliations:** Department of Electrical Engineering, College of Engineering King Khalid University, Abha, Saudi Arabia

**Keywords:** deep convolutional neural networks (CNN), deep learning, ground reaction forces (GRF), gait, interpretable neural networks, Parkinson's disease, perturbation

## Abstract

**Introduction:**

Gait analysis, an expanding research area, employs non-invasive sensors and machine learning techniques for a range of applications. In this study, we investigate the impact of cognitive decline conditions on gait performance, drawing connections between gait deterioration in Parkinson's Disease (PD) and healthy individuals dual tasking.

**Methods:**

We employ Explainable Artificial Intelligence (XAI) specifically Layer-Wise Relevance Propagation (LRP), in conjunction with Convolutional Neural Networks (CNN) to interpret the intricate patterns in gait dynamics influenced by cognitive loads.

**Results:**

We achieved classification accuracies of 98% F1 scores for PD dataset and 95.5% F1 scores for the combined PD dataset. Furthermore, we explore the significance of cognitive load in healthy gait analysis, resulting in robust classification accuracies of 90% ± 10% F1 scores for subject cognitive load verification. Our findings reveal significant alterations in gait parameters under cognitive decline conditions, highlighting the distinctive patterns associated with PD-related gait impairment and those induced by multitasking in healthy subjects. Through advanced XAI techniques (LRP), we decipher the underlying features contributing to gait changes, providing insights into specific aspects affected by cognitive decline.

**Discussion:**

Our study establishes a novel perspective on gait analysis, demonstrating the applicability of XAI in elucidating the shared characteristics of gait disturbances in PD and dual-task scenarios in healthy individuals. The interpretability offered by XAI enhances our ability to discern subtle variations in gait patterns, contributing to a more nuanced comprehension of the factors influencing gait dynamics in PD and dual-task conditions, emphasizing the role of XAI in unraveling the intricacies of gait control.

## 1 Introduction

Gait refers to the distinctive walking pattern unique to each individual (Saleh and Hamoud, 2021). It involves a cyclic sequence of movements in both lower limbs (Jing et al., 2019), providing valuable information about individuals' physical and physiological attributes, including weight, gender, health, and age (Wang and Zhang, 2020; Sadeghzadehyadi et al., 2021).

Gait analysis holds immense importance across various domains, such as healthcare, sport, biometrics, and human–robot interaction. It serves as a rich source of information, adding to the understanding and assessment of various conditions, including neurodegenerative disorders like Parkinson's disease (PD) (Alotaibi and Mahmood, 2015; Yuqi et al., 2019; Chaabane et al., 2023).

Previous studies (Castro et al., 2017; Huang et al., 2021; Wang and Yan, 2021; Erdaş et al., 2022; Vidya and Sasikumar, 2022) have explored gait analysis in the context of PD, aiming to diagnose the condition and track disease progression (Yuan and Zhang, 2018; Zhang S. et al., 2019; Mogan et al., 2023). However, these analyses often rely on clinical evaluation and subjective surveys, resulting in semi-subjective assessments (Wu et al., 2016; Arshad et al., 2021; Khan et al., 2023). Additionally, gait alterations under cognitive load known as “dual tasks” have been investigated, revealing variations influenced by factors such as environmental conditions and emotional states (Delgado-Escaño et al., 2018; Alharthi et al., 2019; Castro et al., 2020; Slijepcvic et al., 2021).

The existing gait analysis in literature faces limitations, particularly in accurately representing the non-linearity and non-stationary of gait cycle (Whittle, 2023). Traditional methods, such as visual observation and harmonic analysis, may fall short of capturing the intricate dynamics of gait (Goodfellow et al., 2016). To address these limitations, this study incorporates explainable artificial intelligence (XAI) techniques. XAI, including layerwise relevance propagation (LRP), enhances the transparency of deep learning models, adding to the interpretation of predictions. We selected LRP over other XAI methods, such as SHAP (SHapley Additive ExPlanations) (Ribeiro et al., 2016), Gradient-weighted Class Activation Mapping (Grad-CAM) (Selvaraju et al., 2017), and Local Interpretable Model-agnostic Explanations (LIME) (Lundberg and Lee, 2017). As noted by Adebayo et al. (2018), not all proposed XAI methods are robust, and the validity of their explanations should be critically assessed.

In this paper, we contribute a comprehensive approach to gait analysis by leveraging sensor fusion, deep convolutional neural networks (CNN), and XAI techniques, specifically LRP. The utilization of CNNs facilitates automatic feature extraction from raw sensor data, while the incorporation of LRP enhances interpretability. This novel combination adds significant value to the fields by providing insights that inform not only gait analysis but also sensor design and data processing for improved healthcare applications.

## 2 Background

### 2.1 Related studies

**Figure 1:**
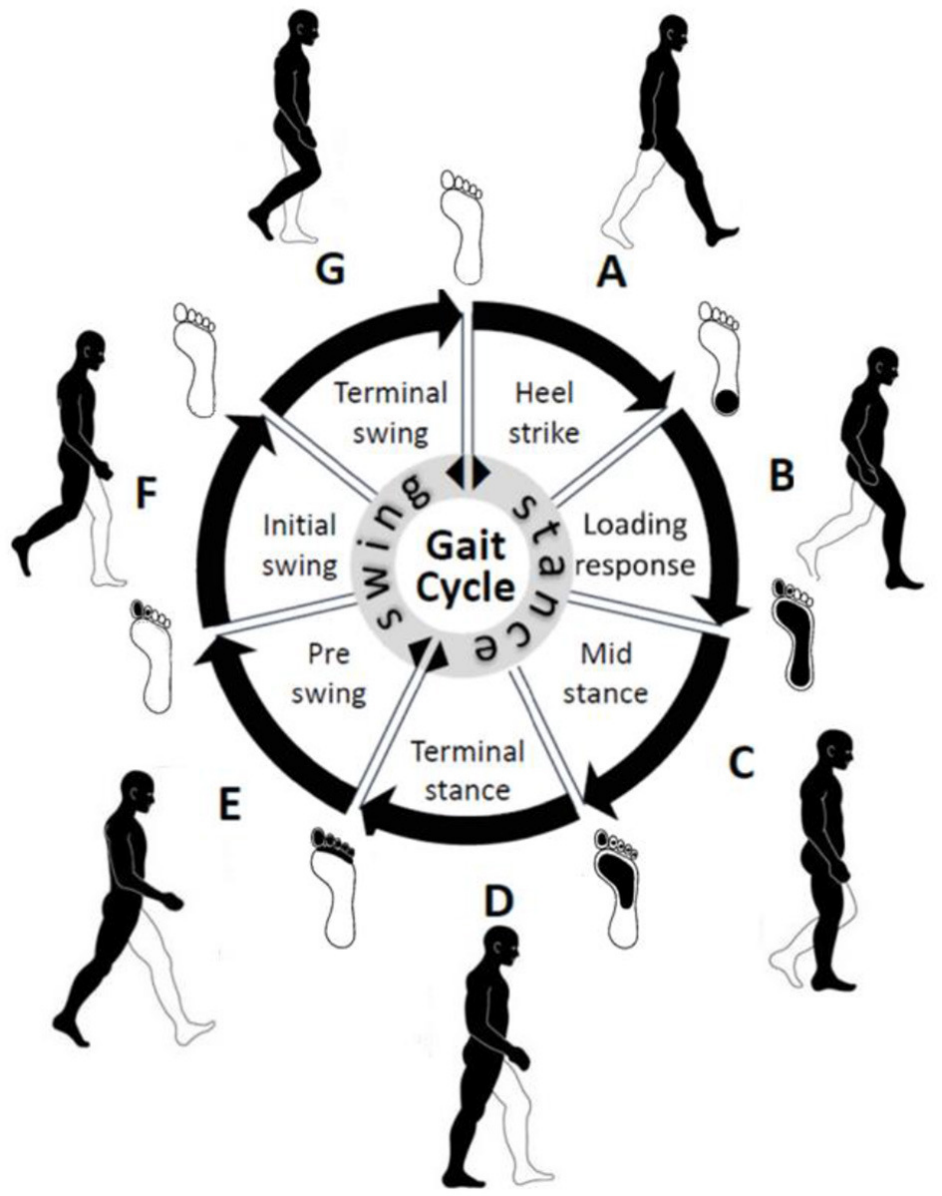
Important gait events and intervals in a normal gait cycle. In the center, the stance phase represents 60% of the gait cycle and the swing phase represents 40% of the gait cycle.

Gait, the intricate walking pattern unique to each individual, has captivated humans (Wang and Zhang, 2020). Figuratively, the gait cycle, as depicted in Figure 1, encapsulates the rhythmic sequence of movement in the lower limb during walking. Early civilizations recognized the distinctiveness of gait as a personal identifier, and over time, methodologies for studying gait have evolved from rudimentary visual observation to sophisticated techniques (Yuqi et al., 2019).

In ancient times, the recognition of individuals based on their gait laid the foundation of contemporary studies (Saleh and Hamoud, 2021). Recent advancements, such as the integration of CNN, have enabled person recognition through intricate gait models (Jing et al., 2019). These efforts underscore the enduring importance of gait analysis, with applications ranging from healthcare to biometrics (Alotaibi and Mahmood, 2015).

The landscape of gait analysis has witnessed a notable surge in recent literature, with cutting-edge technologies at the forefront. For instance, a fusion network incorporating long short-term memory (LSTM) and CNNs demonstrated heightened accuracy in abnormal gait recognition (Sadeghzadehyadi et al., 2021). Another study applied a CNN-LSTM network to decipher spatiotemporal patterns of gait anomalies (Wang and Zhang, 2020), highlighting a continuous evolution of gait analysis methodologies.

Gait biometrics has emerged as a focal point, with studies exploring joint CNN-based methods (Chaabane et al., 2023). Moreover, predicting the severity of neurodegenerative diseases using CNNs showcased promising outcomes (Yuqi et al., 2019). Lightweight attention-based CNN models efficiently recognized gait patterns using wearable sensors, pushing the boundaries of gait analysis capabilities (Alotaibi and Mahmood, 2015). These contemporary studies collectively underscore the growing importance of leveraging advanced technologies for accurate and nuanced gait analysis.

Recent gait recognition literature has focused on solving view- and clothing-invariant problems using advanced machine learning methods like generative adversarial networks (GANs). Zhang P. et al. (2019) designed a view transformation GAN (VT-GAN) with a generator, discriminator, and similarity preserver, achieving competitive results on the CASIA-B dataset. Babaee et al. (2019) used GANs to reconstruct complete gait energy images (GEIs) from incomplete ones, showing effectiveness on the OU-ISIR dataset. Chen et al. (2021) proposed Multi-View Gait GAN (MvGGAN) for cross-view gait recognition, demonstrating improved performance on CASIA-B and OUMVLP datasets. Recent study on wearable and floor sensors has focused on medical applications, such as analyzing the impact of muscle fatigue on gait (Balakrishnan et al., 2020), health monitoring (Muheidat and Tawalbeh, 2020), and age-related differences (Costilla-Reyes et al., 2021). Turner and Hayes (2019) proposed using an LSTM network to classify pressure sensor signals from shoes, aiming to diagnose gait abnormalities. Tran et al. (2021) developed multi-model LSTM and CNN to classify IMU spatiotemporal signals, outperforming previous results on the whuGAIT (Zou et al., 2020) and OU-ISIR (Ngo et al., 2014) datasets.

In the field of gait analysis, the integration of explainable artificial intelligence (XAI) represents a pioneering approach. XAI techniques, exemplified by methods such as layerwise relevance propagation (LRP), address the opacity challenge inherent in deep learning models (Erdaş et al., 2022). LRP has shown success in image classification (Samek et al., 2017a; Jolly et al., 2018) and gait-based subject identification (Horst et al., 2019) when combined with CNNs. Our study stands as a beacon of innovation, presenting a comprehensive approach that seamlessly integrates sensor fusion, CNN, and XAI techniques for gait analysis (Khan et al., 2023).

While existing studies have explored gait analysis through the lens of deep learning models, our distinctive contribution lies in the transparent interpretation facilitated by XAI. Building on recent advancements, we propose using LRP to enhance the interpretability of CNN predictions (Castro et al., 2020). This not only adds intrinsic value to gait analysis but also provides profound insights that extend beyond, influencing advancements in sensor design and data processing for refined healthcare applications (Alharthi et al., 2019). Our study represents a departure from conventional convolutional gait analysis approaches, introducing a paradigm shift in the synergy between gait analysis, deep learning, and explainability.

### 2.2 Gait parameters

Gait refers to the coordinated sequence of muscle contractions that result in walking. The brain generates commands that travel through the spinal cord to activate the lower neural center, leading to muscle contractions aided by feedback from joints and muscles. This allows for coordinated movements of the trunk and lower limbs, resulting in periodic cycles for each foot. These cycles consist of two phases: the stance phase (when the foot is in contact with the ground) and the swing phase (when the foot is not in contact with the ground). The stance phase is further divided into four intervals (A, B, C, and D), while the swing phase is divided into three intervals (E, F, and G) (Whittle, 2023) as shown in Table 1 and Figure 1.

**Table 1:** Gait intervals.

**Sequence**	**Gait interval**	**Description**
A	Heel strike	Initial contact uses this term to describe the contact of the extended limb's heel with the walking surface
B	Loading response	Foot flat is a single-support interval that follows the initial double-support interval. During this phase, the body weight is transferred onto the supporting limb. The trunk is at its lowest position, the knee is flexed, and the ankle undergoes plantar flexion
C	Mid-stance	Single-support interval that occurs between opposite toe-off and heel-off. It commences from the elevation of the opposite limb until both ankles align in the coronal plane
D	Terminal stance	Heel-off begins when the supporting heel rises from the ground in preparation for the opposite swing. During this phase, the trunk is sinking from its highest point, and the knee has an extant peak near the time of heel rise, while the ankle undergoes dorsiflexion after heel rise. The swing phase consists of three intervals: pre-swing, initial swing and mid-swing, and terminal swing
E	Pre-swing	The second double-limb support interval. During this phase, opposite initial contact occurs, and the hip begins to flex, the knee flexes, and the ankle undergoes plantar flexion. The toe is in the last contact before the swing phase, completing the push-off initiated in interval D
F	Initial swing	Mid-swing interval commences with the toe-off into single-support and starting to swing. The body weight shifts to the opposite forefoot, and the knee joint undergoes maximum flexion. The hip flexes, and the limb advances in preparation for a stride
G	Terminal swing	The last interval of the gait cycle and the end of the swing phase. The interval starts at maximum knee flexion and ends with maximum extension of the swinging limb forward. The hip continues to flex while the knee extends with regard to gravity, and the ankle continues dorsiflexion to end neutral, ready for the next heel strike

## 3 Materials and methods

**Figure 2:**
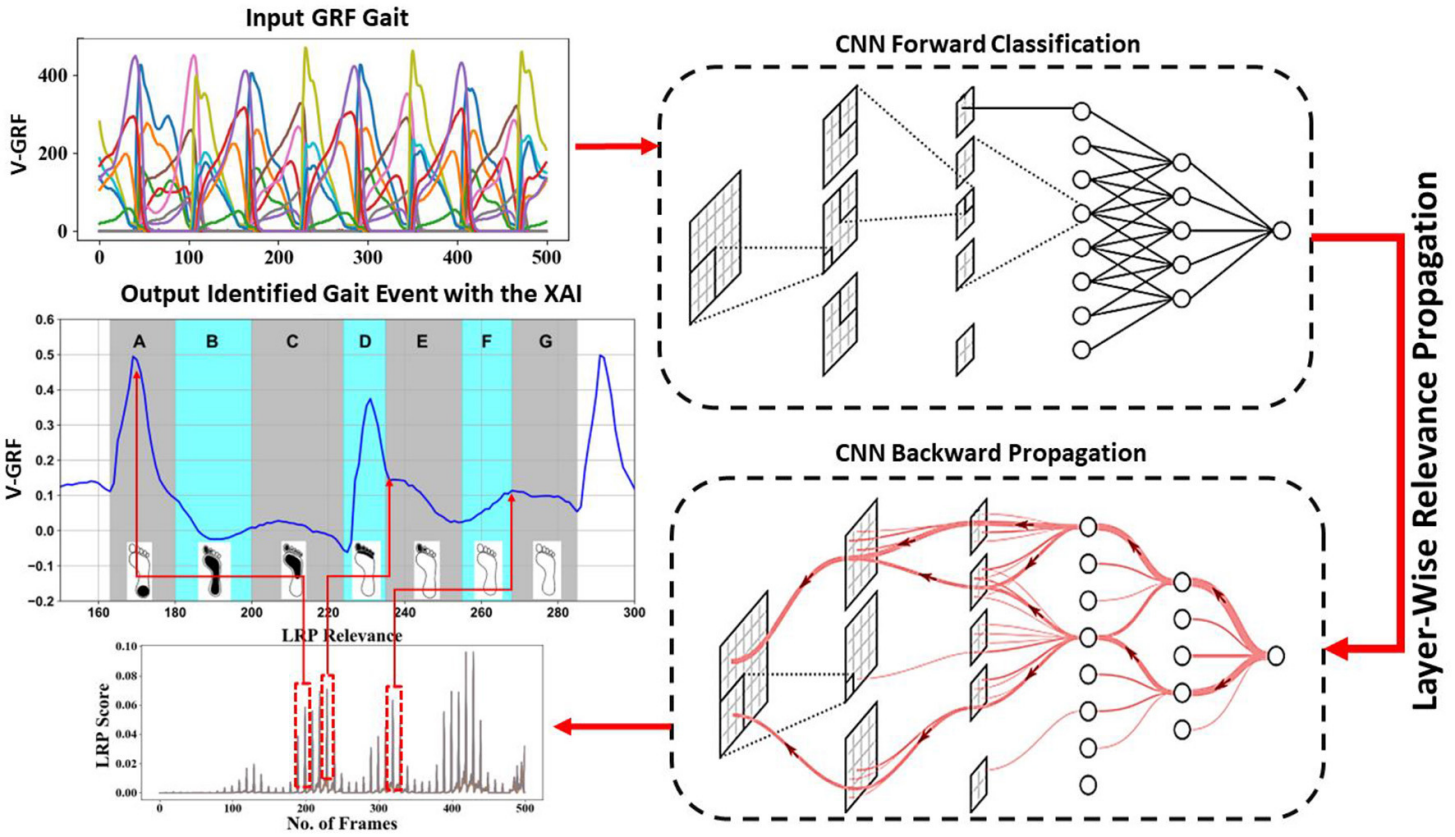
Overview of data acquisition and analysis of CNN. Gait data as input to CNN for classification; interpreting the CNN model by LRP, a deeper red color represents a higher contribution to the classification process. Relevance linked to the foot profile in the input single.

The categorization of gait ground reaction force (GRF) signals poses a formidable challenge, necessitating the application of sophisticated machine learning methodologies. Illustrated in Figure 2, this study delineates the framework for data acquisition and analysis. Gait data presented in Sections 3.6.1 and 3.6.2 serve as the training set for a neural network tasked with classifying these signals, and the resulting output is iteratively refined through backpropagation to pinpoint the key foot profiles crucial for classification. Detailed in subsequent sections are the experiments conducted utilizing various deep convolutional neural network (CNN) models to process and categorize spatiotemporal 3D matrices derived from raw sensor signals.

### 3.1 Convolutional neural networks

CNNs excel in classification tasks by abstracting high-level features from extensive datasets through convolutional operations. Mathematical representation in one-dimensional convolution operations is expressed as *C*(*i*), with *i* denoting the index of an element in the new feature map (Goodfellow et al., 2016, ch. 9):


(1) $$\begin{array}{l} C\left(i\right)=\left(\omega ◦\;x\right)\left[i\right]=\underset{d}{\sum }x\left(i-d\right)\;\omega \left(d\right) \end{array}$$

Gait is captured as a two-dimensional signal as spatial and temporal; therefore, the convolution operation in Equation 1 can be extended to two dimensions, such that the spatiotemporal input is a large set of data points, and the kernel is a set of data smaller in size than the input. Then the convolution operation slides the kernel over the input computes elementwise multiplication and adds the values in a smaller future map. With a 2-D input *x* and a 2-D kernel ω with (*i, j*), (*d, k*) are iterators, the mathematical representation of convolution in two dimensions can expressed as *C*(*i, j*) with (*i, j*) is the index of an element in the new feature map (Goodfellow et al., 2016):


(2) $$\begin{array}{l} C\left(i,j\right)=\left(\omega ◦\;x\right)\left[i,j\right]=\underset{d}{\sum }\underset{k}{\sum }x\left(i-d,\;j-k\right)\omega \left(d,k\right) \end{array}$$

**Figure 3:**
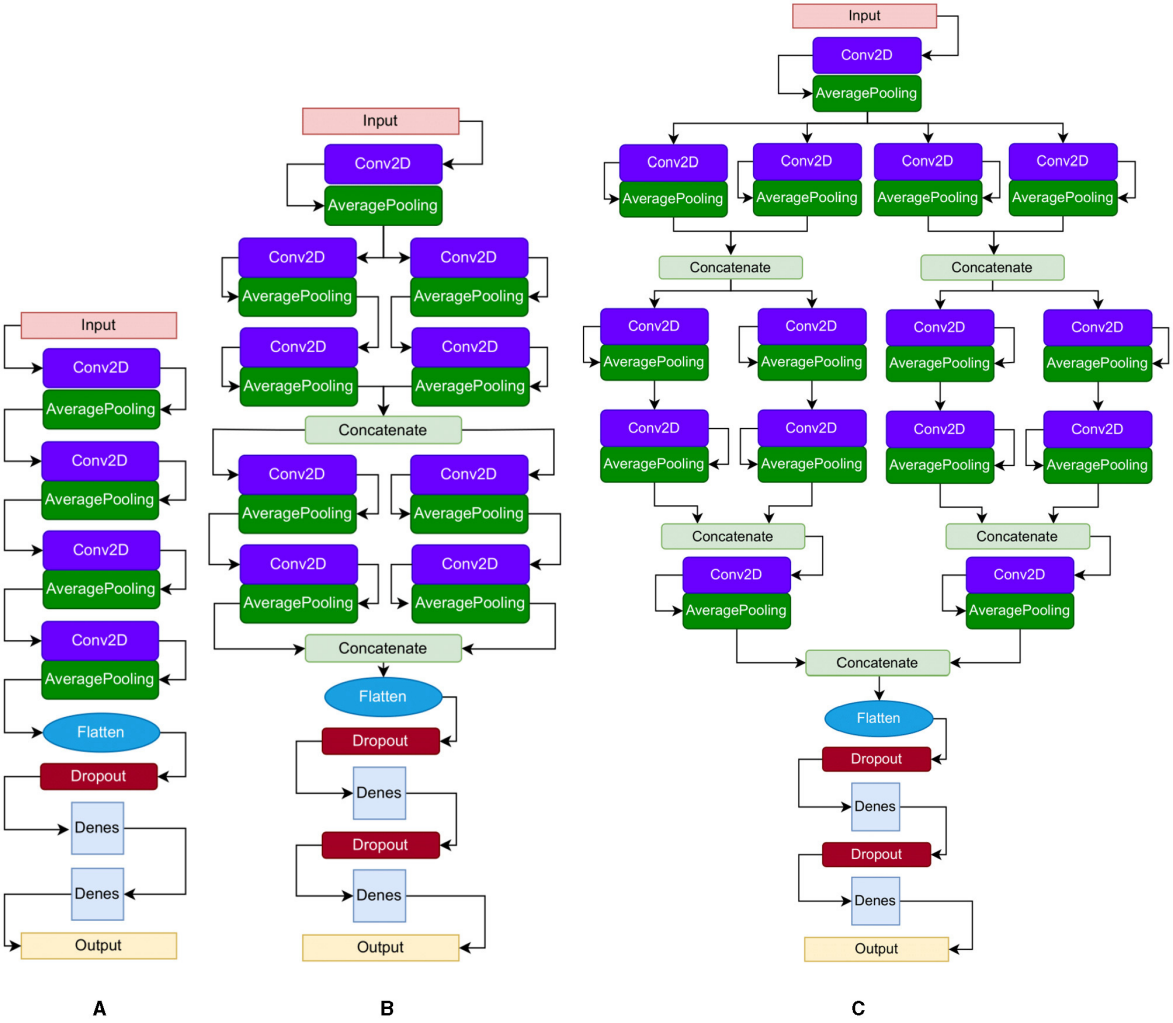
Proposed CNN architectures: **(A)** single CNN, **(B)** parallel CNN, **(C)** quadruplets CNN. The boxes: convolution layers and fully connected layers; pooling layers; concatenation layers and flattening layers; dropout layers.

In this study, we implement three CNN architectures for analyzing gait deterioration. The first model (Figure 3A) is a CNN designed for PD severity classification, comprising four convolutional layers, each followed by average pooling and two fully connected layers, totaling 10 stacked layers. The second CNN architecture (Figure 3B), tailored for processing GRF signals, draws inspiration from inception neural network architectures. It features two stages with parallel streams fused via concatenation layers, resulting in 18 stacked layers. The third CNN (Figure 3C) is a quadruplet network, amalgamating elements from Siamese and triplet networks. It includes convolutional layers, max-pooling, and average pooling, with separate activations, weights, and biases for each stream. This architecture aims to capture spatial and temporal gait signals simultaneously, enhancing generalization capabilities on unseen data.

### 3.2 Backpropagation

It is short for “backward propagation of errors”; it is an algorithm based on gradient descent. As explained by Andrew Ng (Ma et al., 2024), the method moves in reverse order from the output layer to the input layer while calculating the gradient of the error function based on the network weights, the aim is to minimize *J (*θ*)* using an optimal set of parameters in θ. It is based on performing the partial derivative to minimize the cost function. The partial derivative is expressed as $\frac{\partial }{\partial \theta_{i,j}^{l}}\;J\;\left(\theta \right)$. The output layer calculates the error of the network layers *L* with: *d*^(*L*)^ = α^(*l*)^−*y*, such that the error of node *j* in layer *l* is denoted as $d_{j}^{\left(l\right)}$ and the activation of node *j* of layer *l* is denoted as $\alpha_{j}^{\left(l\right)}$ and *y* is the output of the output layer, then the backpropagation can be expressed for neural networks as (Ma et al., 2024):


(3) $${ð}^{\left(L\right)}=({\left(\theta^{\left(l\right)}\right)}^{\left(T\right)}{ð}^{\left(l+1\right)})◦\;\alpha^{\left(l\right)}◦(1-\alpha^{\left(l\right)})$$

Here, the ð* values of the output layer *L* are calculated by multiplying the ð* values in the next layer (in the reverse direction) with the θ matrix of layer *l*; hence, *T* denotes matrix. We then perform elementwise multiply (°) with the *g*′, which is the derivative of the activation function, which is evaluated with the input values given by *z*^(*l*)^, where *g*′(*z*^(*l*)^) = α^(*l*)^°(1−α^(*l*)^).

The partial derivatives needed for backpropagation are performed by multiplying the activation values and the error values for each training example t and m is the number of training data as Ma et al. (2024):


(4) $$\begin{array}{l} \frac{\partial }{\partial \theta_{i,j}^{l}}\;J\;\left(\theta \right)=\frac{1}{m}\;\left\lfloor \overset{m}{\underset{t=1}{\sum }}\alpha_{j}^{\left(t\right)\left(l\right)}{ð}_{j}^{\left(t\right)\left(l+1\right)}\;\right\rfloor . \end{array}$$

### 3.3 Evaluation measure

The confusion matrix is a common accuracy measure in gait analysis (Ruuska et al., 2018). It is a table showing correct and incorrect predictions for each class, including true positive (TP), true negative (TN), false positive (FP), and false negative (F).

In this paper, we use the confusion matrix because a number of TP, TN, FP, and FN samples are values of interest to understand the confusion in gait classes for further analysis using LRP.

From this confusion matrix table, performance measures are obtained, such as accuracy, recall, precision, and F1 using the following equations.

- Accuracy: an indicator of the ratio between the correctly predicted data to the total number of samples in the dataset, defined as follows: $\frac{TP+TN}{TP+TN+FP+FN}$.
- Recall: the proportion of positive classes identified correctly, defined as follows: $\frac{TP}{TP+FN}$.
- Precision: the fraction of positive cases correctly identified over all the positive cases predicted, defined as $\frac{TP}{TP+FP}.$
- F1-Score: the harmonic mean of Precision and Recall, defined as follows: $\frac{2^{*}Precision^{*}Recall}{Precision+Recall}.$

### 3.4 Layerwise relevance propagation

Layerwise relevance propagation (LRP) (Bach et al., 2015; Montavon et al., 2017, 2018) is a backward propagation method used to identify the most influential parts of the input vector in the model prediction of an artificial neural network (ANN). In this thesis, we measure the contribution of individual components of the input *x*_*i*_ (e.g., sensor signals at specific time frames) to the prediction *f*_*c*_*(x)* of a gait class *c* made by the CNN classifier *f*. The prediction is redistributed backward through the network via backpropagation until reaching the input layer. LRP generates a “heat map” over the original signal, highlighting sections with the highest contributions to the model's prediction, such as areas with the greatest variability among classes. It is important to note that a neural network comprises multiple layers of neurons, where neurons are activated as described in Montavon et al. (2018).


(5) $$a_{k}=\sigma (\sum_{j}a_{j}\omega_{jk\;}+b_{k})$$

Here, *a*_*k*_ is the neuron activation and *a*_*j*_ is the activation of the neuron in the previous layer in a forward direction; ω_*jk*_ denotes the weight received in the forward direction by neuron *k* from neuron *j* in the previous layer, and *b*_*k*_ is the bias. The sum is computed over all the *j*^th^ neurons that are connected to the *k*^th^ neuron. σ is a non-linear monotonically increasing activation function. These activations, weights, and biases are learned by CNN during supervisory training. During training, the output *f*_*c*_(*x*) is evaluated in a forward pass and the parameters (ω_*jk*_+ *b*_*k*_) are updated by back-propagating using model error. For the latter, we base our computations on categorical cross-entropy (Zhang and Sabuncu, 2018).

The LRP approach decomposes the CNN output for a given prediction function of gait class *c* as *f*_*c*_ for input *x*_*i*_ and generates a “relevance score” *R* for the *i*th neuron received from *R*_*j*_ for the *j*th neuron in the previous layer, which is received from *R*_*k*_, for the *k*th neuron in the lower layer, where the relevance conservation principle is satisfied as:


(6) $$\begin{array}{l} \underset{i}{\sum }R_{i\leftarrow j}=\;\underset{j}{\sum }R_{j\leftarrow k}=\underset{k}{\sum }R_{k}\;=f_{c}\left(x\right) \end{array}$$

The LRP starts at the CNN output layer after removing the *Softmax* layer. In this process, a gait class *c* is selected as an input to LRP, and the other classes are eliminated. The backpropagation for unspooling for the pooling layer is computed by redirecting the signal to the neuron for which the activation was computed in the forward pass. As a generalization, consider a single output neuron *i* in one of the model layers, which receives a relevance score *R*_*j*_ from a lower-layer neuron *j*, or the output of the model (class *c*). The scores are redistributed between the connected neurons throughout the network layers, based on the contribution of the input signals *x*_*i*_ using the activation function (computed in the forward pass and updated by back-propagating during training) of neuron *j* as shown in Figure 2. The latter will hold a certain relevance score based on its activation function and pass its value to consecutive neurons in the reverse direction. Finally, the method outputs relevance scores for each sensor signal at a specific time frame. These scores represent a heat map, where the high relevance scores at specific time frames highlight the areas that contributed the most to the model classifications.

### 3.5 Perturbation analysis

Human gait, characterized by its inherent variability among individuals and even within a single individual, poses a significant challenge for developing reliable and robust models capable of accommodating such diversity in input data. Within the realm of gait analysis, layerwise relevance propagation (LRP) emerges as a promising methodology for interpreting the significance of input data points. However, the effectiveness of LRP in the context of gait analysis hinges on its resilience to noise and fluctuations in the input data stream.

To address this concern, a systematic exploration of the impact of random perturbation noise on LRP relevance scores is undertaken. This analysis serves a dual purpose: first, to inform the selection of the most appropriate LRP method, and second, to guide the design of a deep convolutional neural network (CNN) model capable of withstanding the inherent variability of gait patterns. The intricacies of this perturbation analysis methodology are elucidated in subsequent sections.

The iterative procedure proposed by Samek et al. (2017b), commonly referred to as the “greedy” approach, serves as the cornerstone for selecting the optimal LRP method and evaluating the relevance scores generated for gait classification. This iterative process involves progressively removing information from the spatiotemporal input signal, prioritizing regions with the highest relevance scores for perturbation using a “most relevant first” (MoRF) approach (Samek et al., 2017b). At each iteration, the model's performance is rigorously assessed by re-predicting test data with the accumulated perturbations. The selection of the preferred LRP method is informed by observing the most significant decline in accuracy during the initial iterations, indicating the criticality of the perturbed regions for accurate classification performance. Subsequent iterations demonstrate a slower decline in accuracy as less crucial regions are perturbed, thus providing insight into the relative importance of different input features.

Moreover, the evaluation of the significance of CNN model architecture entails a comprehensive analysis of the impact of perturbations on model performance. This process involves systematically removing the highest relevance scores obtained from the selected LRP method and evaluating the model's performance by re-predicting the test data for each perturbed model. Models exhibiting substantial performance deterioration after only a few perturbation steps are deemed most amenable to leveraging LRP. This decline in performance signifies the critical role of the removed regions in facilitating accurate classification, thereby highlighting meaningful relationships between input patterns and learned classes. Conversely, regions with minimal impact on classification performance upon removal suggest lesser relevance in discerning such relationships, thus informing subsequent model refinement efforts.

### 3.6 Gait data

In this paper, we investigate gait deterioration due to Parkinson's disease (PD) and under dual-task conditions (walking while performing cognitive tasks as detailed in Section 3.6.2). Specifically, we compare the effects of dual-tasking and PD on gait events. The data for this study are detailed in the following section.

#### 3.6.1 Parkinson's disease data

In this study, we utilized the open access benchmark available on PhysioNet.org (Goldberger et al., 2003) to analyze ground reaction force (GRF) data in Parkinson's disease (PD) patients. The dataset included 93 PD patients (mean age: 66.3 years; 63% men) with varying degrees of PD progression based on Hoehn and Yahr Scale staging criteria (Frenkel-Toledo et al., 2005; Yogev et al., 2005; Hausdorff et al., 2007), as outlined in Table 2, and described in detail in Table 3. Additionally, the dataset also included GRF measurements from 73 healthy controls (mean age: 66.3 years; 55% men). During the data collection process, participants were instructed to walk for ~2 min while wearing eight sensors placed underneath each foot to measure the force [N] as a function of time. The output of the 16 sensors was recorded at a frequency of 100 frames per second. Moreover, the sum of the eight sensors of each foot was added to each subject sample along with the timestamp, resulting in a total of 19 columns. The dataset was collected by three research groups, namely the Ga group (Yogev et al., 2005), the Ju group (Hausdorff et al., 2007), and the Si group (Frenkel-Toledo et al., 2005). The sub-parts of the dataset were named after these research groups. The Ju and Si groups recorded usual healthy walking at a self-selected speed, while the Ga group included additional samples for each subject, where they performed a dual task while walking (Yogev et al., 2005). Overall, this dataset provides valuable insights into the gait patterns of PD patients and healthy individuals, which could be used to develop effective interventions for gait-related impairments in PD.

**Table 2:** Number of subjects with the severity rating.

**Severity (0) healthy**	**Severity (2)**	**Severity (2.5)**	**Severity (3)**	**Group**
18	15	15	6	Ga (Balakrishnan et al., 2020)
26	12	12	4	Ju (Muheidat and Tawalbeh, 2020)
29	29	29	0	Si (Costilla-Reyes et al., 2021)

**Table 3:** Discerption of datasets subject.

**Subjects**	**Number**	**Male**	**Female**	**Group**
PD patients	29	20	9	Ga (Balakrishnan et al., 2020)
Healthy subjects	18	10	8	Ga (Balakrishnan et al., 2020)
PD patients	29	16	13	Ju (Muheidat and Tawalbeh, 2020)
Healthy subjects	26	12	14	Ju (Muheidat and Tawalbeh, 2020)
PD patients	35	22	13	Si (Costilla-Reyes et al., 2021)
Healthy subjects	29	18	18	Si (Costilla-Reyes et al., 2021)

**Figure 4:**
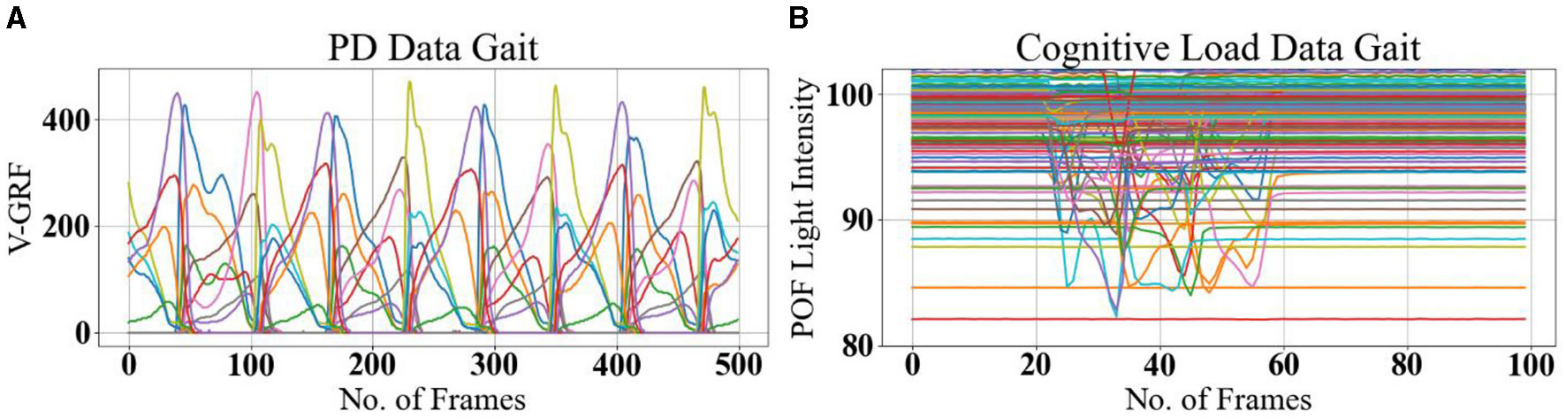
Example gait data. **(A)** PD Gait was recorded at 100 frames per second, with a sample length of 500 timeframes. The signals represent pressure sensor signals under each foot (different colors for each of the eight sensors). **(B)** Cognitive Load Gait, recorded at 20 frames per second, with a sample length of 100 timeframes. The signals represent POF sensors transmitted light intensity is affected by surface bending due to pressure under each foot (different colors for each of the 116 POF sensors).

Each sample recorded in the dataset contains 19 columns of data with varying column lengths, as for some subjects' gait was recorded for a longer time (12,119 frames) than for others (< 1,000 frames). In order to make the input data length consistent, the datasets were split into equal-size parts of 500 frames such that single long recordings are divided into several chunks of 500 frames. The timestamp columns were deleted as it doesn't report information about gait. The final sample size is 18 columns and 500 rows or frames as shown in Figure 4A. This choice is justified as the gait cycle is ~1 s, and the sample captures heel strike and toe-off for both feet over five gait cycles. The input dataset is a tensor with dimensions *m* × 500 × 18 where *m* = 2,698 for the Ga group (Yogev et al., 2005), 2,198 for the Ju group (Hausdorff et al., 2007), and 1,509 Si group (Frenkel-Toledo et al., 2005). Data standardization is performed as a pre-processing step to reduce the redundancy and dependency among the data, such that the estimated activations, weights, and biases will update similarly, rather than at different rates, during the training process. The standardization involves rescaling the distribution of values with mean at zero and rescaling the standard deviation to unity.


(7) $$\begin{array}{l} \hat{x_{n,s}}=\frac{x_{n,s}-\mu \left(x_{n,s}\right)}{\vartheta \left(x_{n,s}\right)} \end{array}$$

Here, $\hat{x_{n,s}}$ is PD data rescaled such that μ is the mean values and ϑ is the standard deviation. Then, the dataset is randomly split into training 60%, hold-out validation 20%, and testing 20% with a *random state* parameter with a different seed.

#### 3.6.2 Cognitive load data

The iMagiMat footstep imaging system is an innovative floor sensor head that utilizes photonic guided-path tomography technology (Ozanyan et al., 2005; Cantoral et al., 2011; Cantoral-Ceballos et al., 2015; Ozanyan, 2015). The system can capture temporal samples from strategically placed distributed POF sensors on top of a deformable underlay of a commercial retail floor carpet in an unobtrusive manner. Each sensor is made up of low-cost POF (step-index PMMA core with fluorinated polymer cladding and polyethylene jacket, total diameter 1 mm, NA = 0.46) terminated with an LED (Multicomp OVL-3328 625 nm) at one end and a photodiode (Vishay TEFD4300) at the other. The sensors are designed to allow collaborative sensor fusion and deliver spatiotemporal sampling that is adequate for discerning gait events.

The iMagiMat system covers a 1 m × 2 m area managed by 116 POF sensors arranged in three parallel plies, sandwiched between the carpet top pile and the carpet underlay. The system includes a lengthwise ply with 22 POF sensors at 0° angle to the walking direction and two independent plies, each consisting of 47 POF sensors, arranged diagonally at 60 and −60°, respectively (see Cantoral-Ceballos et al., 2015, for the iMAGiMAT system). The system is managed by electronics contained in a closed hard-shell periphery at carpet surface level and is organized into eight-channel modules, including LED Driver boards and input trans-impedance amplifier boards to receive the data and send it to a CPLD (complex programmable logic device) to reformat the data for processing by a Raspberry pi single-board computer for export via Ethernet/Wi-Fi. The operational principle of the system is based on recording the deformation caused by the variations of ground reaction force (GRF). As bending affects the POF sensors, transmitted light intensity is affected by surface bending. This captures the specifics of foot contact and generates robust data without constraints of speed or positioning anywhere on the active surface.

For this experiment, 21 physically active subjects aged 20–40 years, 17 men and four women, without gait pathology or cognitive impairment, participated. The study was carried out under the University of Manchester Research Ethics Committee (MUREC) with ethical approval number 2018-4881-6782. All participants were informed about the data recording protocol according to the ethics board's general guidelines, and written consent was obtained from each subject prior to the experiments. Each participant was asked to walk normally or while performing cognitively demanding tasks along the 2 m length direction of the iMagiMat sensor head. The captured gait data was unaffected by start and stop, as it was padded on both ends with several unrecorded gait cycles before the first footfall on the sensor. With a capture rate of 20 timeframes/s (each timeframe comprising the readings of all 116 sensors), experiments yielded 5 s long adjacent time sequences, each containing 100 frames. The recorded gait spatiotemporal signals were able to capture ~4–5 uninterrupted footsteps at each pass.

A dual-task gait test detects mild cognitive impairment (Wang et al., 2023); therefore, five manners of walking were defined as normal gait plus four different dual tasks, and experiments were recorded for each subject, with 10 gait trials for each manner of walking in a single assessment session. Thus, the total number of samples is 10 × 5 = 50 per-subject. The five manners of walking are defined in Table 4. A set of measured data as $x_{n,s}=\left[\begin{array}{c} x_{n,1}\&\ldots \&x_{n,116} \end{array}\right]\in {\mathbb{R}}^{n\times 116}$is harvested from the iMagiMat system, where *n* is the number of the data block (100 frames) and *s* enumerates the POF sensors, as shown in Figure 4B. A total number of 1, 050 samples are recorded for 21 subjects and placed in a 3*D* matrix of dimensions 1, 050 × 100 × 116. The recorded amplitude of data varies due to the weight of each subject; therefore, data standardization is implemented as a pre-processing step, to ensure that the data are internally consistent, such that the estimated activations, weights, and biases update similarly, rather than at different rates, during the training process and testing stage. The standardization involves rescaling the distribution of values with a zero mean unity standard deviation, using Equation 7, where $\hat{x_{n,s}}\;$is gait data rescaled so that μ is the mean and ϑ is the standard deviation. Then, the dataset is randomly split into training 60%, hold-out validation 20%, and testing 20% with a *random state* parameter with a different seed.

**Table 4:** Cognitive load experiment data.

**Manner of walking**	**Description**
M1	Normal Gait: walking at a normal self-selected speed
M2	Gait while listening to a story: audio input through headphones, followed by answering questions after gait recording is completed
M3	Gait with serial 7 subtractions: normal walking speed attempted while simultaneously performing serial 7 subtractions (counting backward in sevens from a given random 3-digit number)
M4	Gait while texting: normal walking speed attempted while simultaneously typing text on a mobile device keyboard
M5	Gait while talking walking at a normal self-selected speed while talking or answering questions

## 4 Experiment and results

All algorithms for LRP computation are implemented in *Python* 3.7.3 programming language using *Keras* 2.2.4, *TensorFlow* 1.14.0, *and iNNvestigate GitHub repository* (Alber et al., 2018). The codes are executed on a desktop with *Intel Core i*7 6700 *CPU @*3.4 *GHz*. The deep CNN model is applied to the datasets to test the validity of the algorithms for identifying gait signatures. The implementation and the perturbation analysis are detailed in the following section. We compare the CNN predictions to manually labeled ground truth in several experiments, including PD severity staging, individuals' identity, and the effects of cognitive load on normal gait. The models' classification performance is evaluated using confusion matrices. The performance of the LRP methods is examined in detail in the discussion subsection.

### 4.1 Classification experiments

We introduce a variety of algorithms and architectures, including a CNN model, LSTM, Stochastic Gradient Descent (SGD), K-Nearest Neighbors (KNN), and Gaussian Process Classifier (GPC). The SGD updates model parameters iteratively using a single or a few randomly selected data points to compute the gradient, optimizing the objective function efficiently for large datasets (Zhang, 2004). The KNN algorithm employs Euclidean distance techniques to determine the distance between data samples (Altman, 1992). The GPC leverages Gaussian processes to define a distribution over functions, making predictions by averaging over all possible functions, thus providing probabilistic classification outputs and well-calibrated uncertainties (Xiao et al., 2019). Through experimentation detailed in the following sections, we utilize CNN and LSTM methods as automatic feature extractors and classifiers. The CNN models shown in Figure 3 map the gait spatiotemporal signal $\hat{x_{n,s}}$ to an output label *y* by learning an approximation function $y\;=\;f\;\left(\hat{x_{n,s}}\right)$. The networks consist of an input layer, convolution layers (see Equation 2), pooling layers, fully connected layers, batch normalization layers, and an output layer with a softmax classifier. The set of stacked layers in Figure 3 utilizes Conv2D kernels (*filter size* × *number of feature maps* × *number of filters*), MaxPooling and AveragePooling layers.

To improve the model performance, a regularization method is utilized as follows: (1) Batch normalization [to normalize the activations of the previous layer at each batch, by maintaining the mean activation close to 0 and the activation standard deviation close to 1 (Ioffe and Szegedy, 2015)]. (2) The Batch normalization followed by dropout (Srivastava et al., 2014), after the last pooling layers were flattened, by transforming a matrix to one single-column vector. An Adam (adaptive moment estimation) (Kingma and Ba, 2015) is utilized to train the model. The used optimizer parameters are α = 0.002, β1 = 0.9, β 2 = 0.999, ε = 1*e*−08. Here, α is the learning rate or the fraction of weights updated where larger values (e.g., 0.3) result in faster initial learning before the rate is updated. Smaller values (e.g., 1.0E-5) slow learning right down during training; β 1 and β 2 are the exponential decay rates for the first- and second-moment estimates, respectively; ε is a small number to avoid division by zero. The loss is computed using categorical cross-entropy in every iteration to minimize the network error (Zhang and Sabuncu, 2018). The convolutional layers weight parameters are initiated with a *Glorot* uniform (Glorot and Bengio, 2010) with zero bias. The model is trained and validated (for several experiments) using a batch size of 100 samples for each iteration; 200 epochs are found optimal to train the model based on backpropagation Equations 3 and 4. The training and validation sizes are set to be 70 and 10%, respectively, where 20% is reserved for testing the model accuracy.

#### 4.1.1 Experiment (1) on PD severity staging

**Figure 5:**
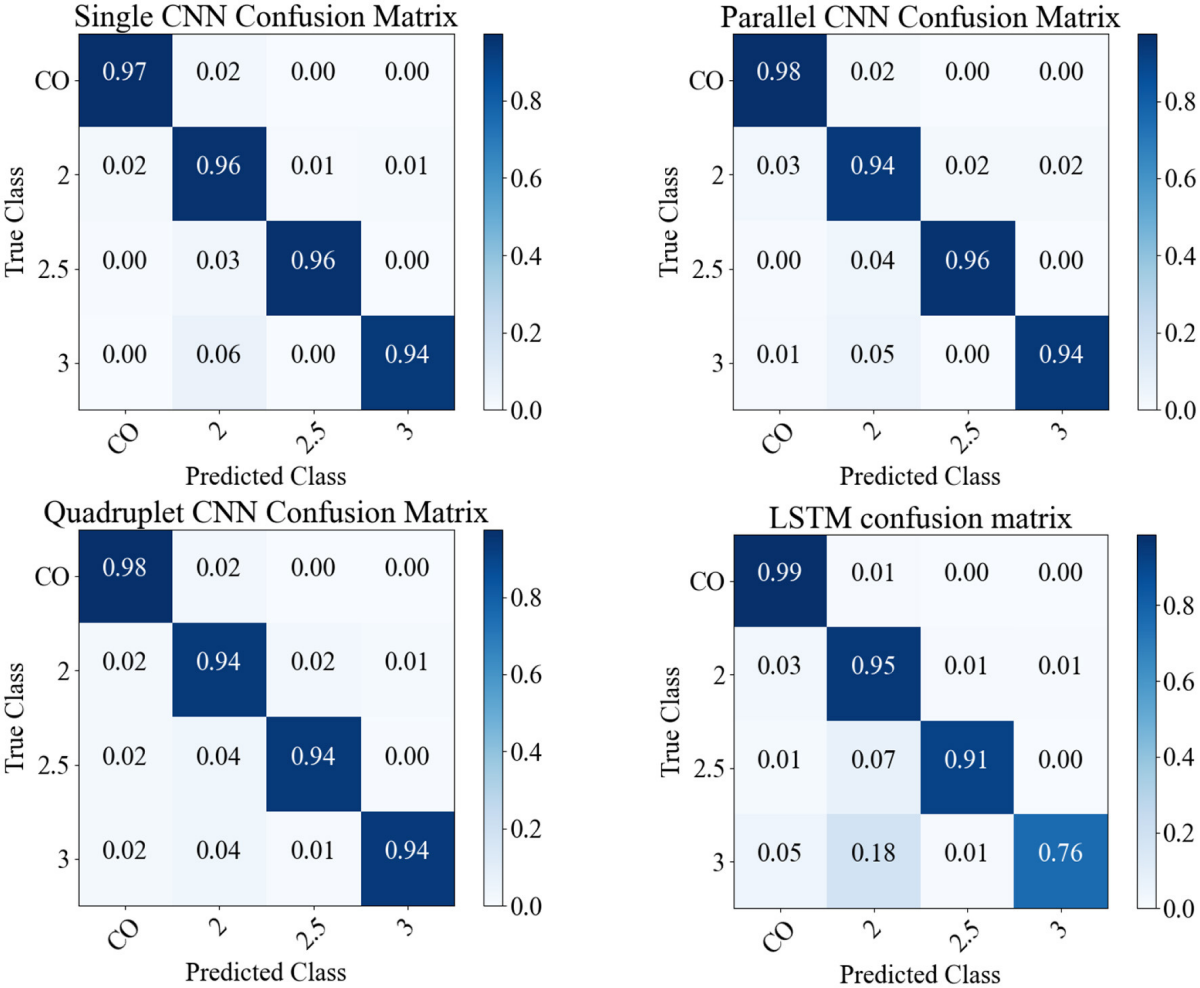
The predictions of models on the 1,281 sample shown as confusion matrices: single CNN, parallel CNN, quadruplet CNN, and LSTM.

In this experiment, CNNS, LSTM, SGD, KNN, and GPC models are trained and tested on the PD dataset to classify the severity of PD into five stages: normal (*CO*), mild (2), moderate (2.5), and severe (3). Table 5 presents the models' *F*1-score for each dataset and the *F*1-score with datasets combined. Figure 5 presents the confusion matrix for the CNNs and LSTM with datasets combined. The best performance is achieved by the CNN single and parallel with F1-score 96% for the data set combined and for each data, where the LSTM performance was 79% for PD stage 3. In the statistical analysis, the performance of SGD, KNN, and GPC models was below 90%.

**Table 5:** PD data models F1-score for each dataset and F1-score with datasets combined.

**CNN model**	**Ga**	**Ju**	**Si**	**GaUJuUSi**
Single	**98%**	**98%**	**98%**	**96%**
Parallel	96%	97%	96%	**96%**
Quadruplet	97%	97%	98%	95%
LSTM	91%	93%	80%	94%
SGD	88%	84%	80%	83%
KNN	81%	90%	78%	79%
GPC	82%	85%	89%	81%

The best performance is in bold.

#### 4.1.2 Experiment (2) on cognitive load impact on gait

**Figure 6:**
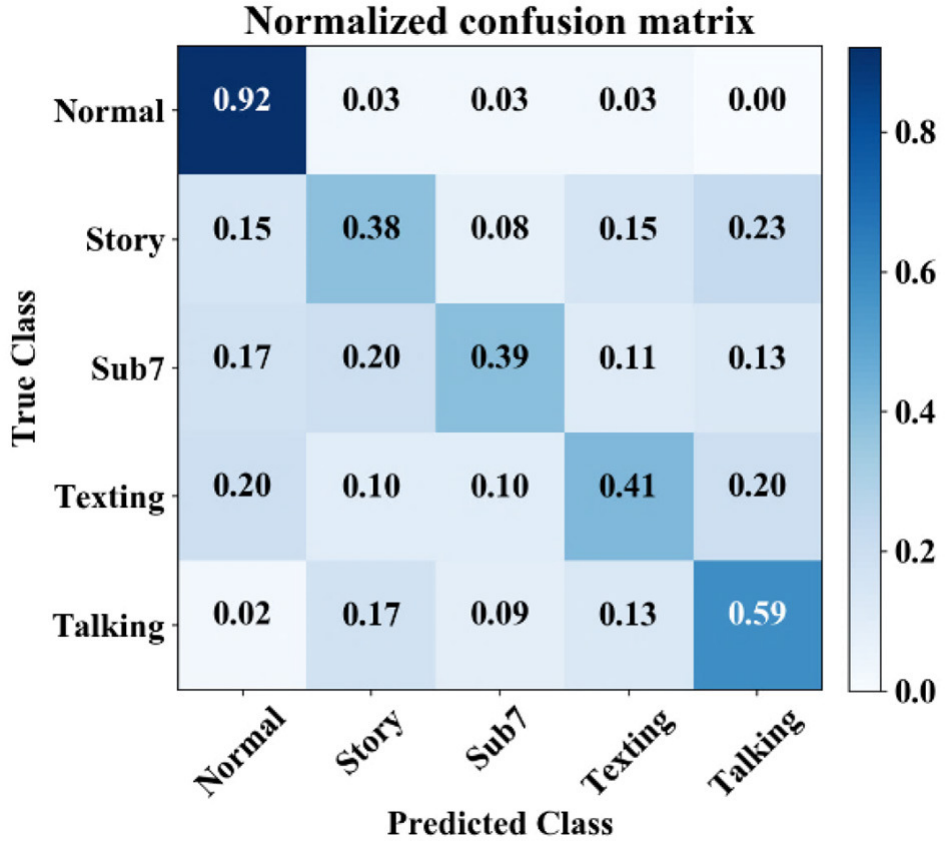
Confusion matrix for classification under cognitive load: 21 subjects, five classes. Experiment (4) Cognitive Load Impact on Gait Variations (Binary Classification).

The aim of this experiment is to show that in healthy subjects the influence of cognitive load on gait varies from subject to subject and the normal gait can be predicted with higher true positive rates than predictions under cognitive load. Five types of gait signatures, normal and four cognitively demanding task patterns, are learned for 21 subjects. The performance observed for the five classes is shown in Figure 6, as the median confusion matrix based on several runs with the CNNs in Figure 3 resulted in a *F*1-score of 50%, mean performance, and standard error of 48.25 ± 1.03%. The results show that normal gait is predicted by a true positive incidence of 92%±1.7%, while there is notable confusion between the dual tasks performed by the 21 subjects. The different random state parameters return the same result, where the normal gait true positive prediction is higher than 90% and substantial confusion between the dual-task cases.

#### 4.1.3 Experiment (3) cognitive load impact on gait for each subject

In this experiment, gait patterns are investigated within each subject, to show that each subject gait under cognitive load can be learned and predicted. This is achieved by training, validating, and testing the CNNs in Figure 3 to classify each subject gait pattern using the normal gait and cognitive load. Each subject data are split using a random state to cover all five classes for testing with *m* = 50 samples. The model evaluation using the *F*1-score is detailed for each subject in Table 6. Gait data are predicted with more than 85% *F*1-score for 16 subjects, and for six subjects, *F*1-scores are between 65 and 77%.

**Table 6:** Models classification accuracy for cognitive load impact on gait for each subject.

**Subject number**	**F1-score**	**Subject number**	**F1-score**	**Subject number**	**F1-score**
0	95%	7	87%	14	100%
1	65%	8	90%	15	75%
2	93%	9	90%	16	80%
3	90%	10	77%	17	100%
4	87%	11	91%	18	100%
5	91%	12	90%	19	80%
6	73%	13	100%	20	69%

#### 4.1.4 Experiment (4) cognitive load impact on gait for each subject

To study patterns for each of the four dual tasks (*M*2−*M*5) representing variants of cognitive load, we organize the data into four groups so that binary classification performance to distinguish between gait under normal (class 0) and cognitive load (one of the classes 1, 2, 3, or 4, depending on the particular data group) conditions can be studied separately for each dual task. The CNNs in Figure 3 are trained 16 times, implementing four runs with each of the four data groups. The *F*1-scores for each run are shown in Table 7. The first run in each data group is based on training and validating the CNNs on 20 subjects and testing the model on 1 subject, to see whether we can predict the gait of one person from 20 people. In the second run, the numbers are 19 and 2, respectively; in the third—17 and 4, respectively. The last run is based on splitting the data into 70% for training, 10% for validation, and 20% for testing, using *m* = 420 samples with a random state of 200 seed parameters (as the accuracy does not change with the random state seed). As shown in Table 7, the highest classification performance is achieved in the first runs (except for the group containing class 3). This is used essentially in the implementation of LRP to analyze the gait classes for that subject in the first run as reported in further comparison with statistical classifiers.

**Table 7:** F1-score predictions for binary classification, normal vs. cognitive load.

**Data group for classification**	**1 testing subject**	**2 testing subjects**	**4 testing subjects**	**Test with all subjects**
Class 0 vs. class 1	100%	85%	81%	79%
Class 0 vs. class 2	95%	87%	58%	69%
Class 0 vs. class 3	60%	68%	63%	79%
Class 0 vs. class 4	100%	85%	74%	81%

### 4.2 LRP analysis and interpretation for explainability

In the following sections, we present the LRP analysis (see Equations 5 and 6) and interpretation for the best-performing model using perturbation presented in Section 3.5. Then, we present the explainability results of the investigated classification models for PD and cognitive load.

#### 4.2.1 Model selection and XAI selection

##### 4.2.1.1 Model selection

**Figure 7:**
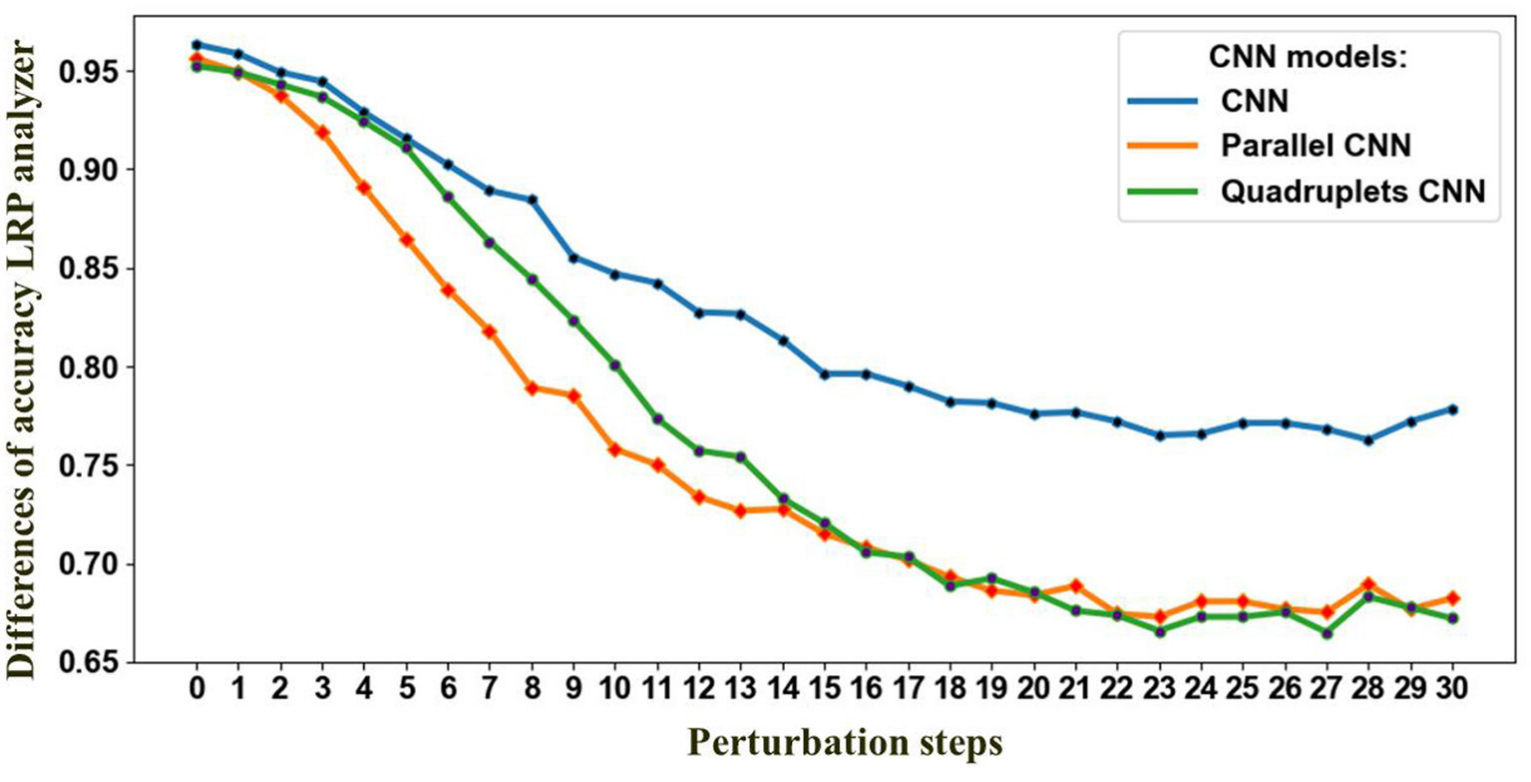
Perturbation effect on the proposed CNNs architectures. The decline in accuracy results from progressively removing information from the input data based on LRP-SPF and re-predicting, at each step, 30 steps total.

**Figure 8:**
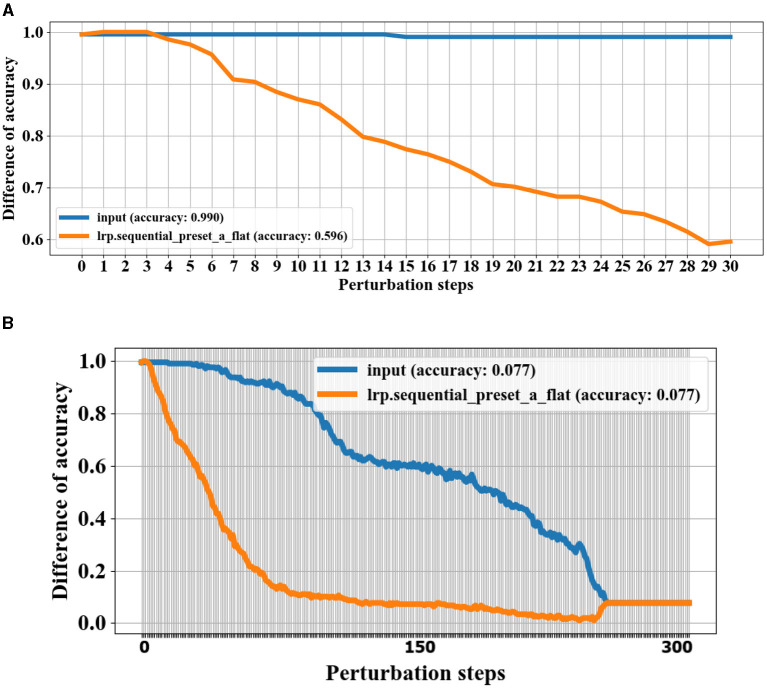
Validation of LRP heatmaps by perturbation technique for experiment 3 subject 13. Information with the highest relevance scores is progressively removed, and the test samples are re-predicted. A steeper initial decrease indicates better identification of gait events with the most weight in the classifications. **(A)** Shows the model predictions in 30 steps based on removing relevance scores using LRP sequential preset a flat (LRP-SPF) and random removal of information. **(B)** Shows the model performance after 300 steps of information removal.

In this study, we conducted an in-depth analysis of the performance of various CNN models for the task of gait classification. We employed explainable AI (XAI) techniques to select the most suitable CNN model for this application. To identify the CNN model that best captures the relevant gait features, we utilized a perturbation-based approach presented in Section 3.5. Specifically, we systematically perturbed each of the three candidate CNN models by gradually replacing 7 × 7 regions within the input gait sequence with Gaussian noise and observed the impact on the classification accuracy over 100 steps. Rather than comparing the models to a baseline, we focused on the rate of decline in accuracy (with the means removed to isolate the rate of change) as a metric to identify the model with the steepest drop in performance. This approach is based on the premise that models that rely on more compact regions within the gait cycle sequence will exhibit a faster decline in accuracy when those regions are perturbed. The results, as depicted in Figure 7, show that the parallel CNN model (see Figure 7) experiences the most pronounced decrease in accuracy with perturbation, indicating that it captures the gait events that are most vulnerable to deterioration in individuals with PD. As depicted in Figure 7, after step 13, the quadruplet CNN begins capturing less relevant features, similar to the decline observed in the parallel CNN. This finding suggests that the parallel CNN model is the preferred candidate for accurate feature identification of the gait cycle events most sensitive to the effects of either PD or cognitive load. Figure 8 shows the assessment of the validity of the LRP heatmaps for subjects' identification of cognitive load.

Here, we apply the removal of the region based on both LRP sequential preset a flat (LRP-SPF) MoRF and random region removal and re-predicting gait class. As shown in Figure 8A, the model prediction strongly decays using the LRP for the removal of information compared to the removal of random information. Figure 8B shows the model performance over 300 steps. The model reaches the lowest performance accuracy where the gait classes have to take a random prediction. Furthermore, it can be inferred from Figure 8 that the model is effective in finding the most relevant region to identify cognitive load of subjects and the LRP is consistent over the test samples.

##### 4.2.1.2 XAI selection

**Figure 9:**
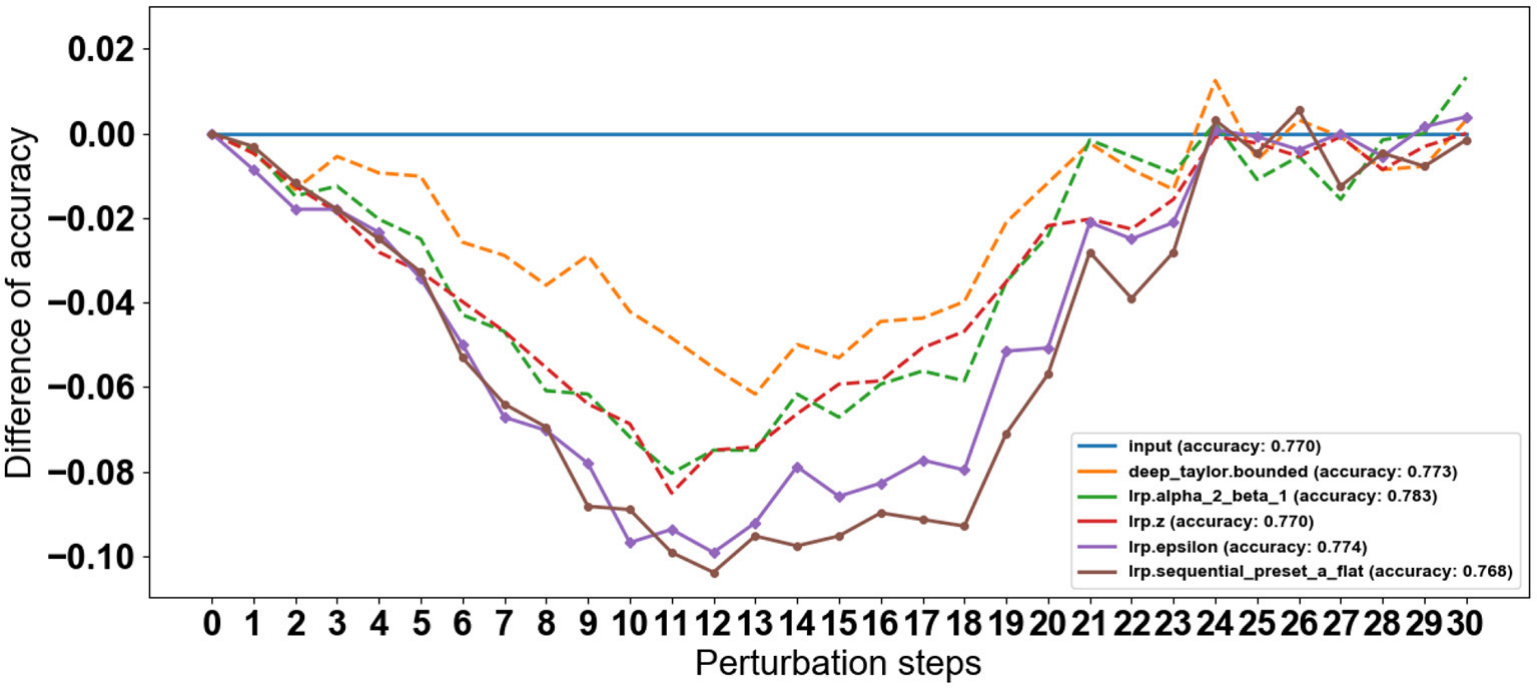
LRP method selection by perturbation steps progressively removes information with the highest relevance scores. A steeper initial decrease indicates better identification of gait events with the most weight in the classifications.

To identify the most suitable backpropagation method for the three CNN models, we conducted a comprehensive evaluation of various LRP (layerwise relevance propagation) techniques. These included deep Taylor (Montavon et al., 2017), deep Taylor bounded (Kohlbrenner et al., 2019), deconvnet (deconvolution) (Zeiler and Fergus, 2014), guided backprop (guided backpropagation) (Springenberg et al., 2015), and LRP sequential preset a flat (LRP-SPF) (Kohlbrenner et al., 2019), all of which were implemented using the iNNvestigate GitHub repository. For each of the LRP methods, we assessed the CNN classification accuracy by performing a sequence of perturbation steps as described in Section 3.5 as described in **Model Selection**. To establish a baseline for comparison, we replaced regions of the input data with random Gaussian noise with one level at 0.1%, rather than using the LRP-based methods. We then subtracted the accuracy of LRP maps from the accuracy of randomly replaced regions to isolate the impact of the LRP techniques. As shown in Figure 9, the LRP curves recovered after around the 15th perturbation step as the remaining spatiotemporal regions became less relevant for the classification task. The baseline accuracy was reached around the 30th perturbation step, indicating that the remaining regions were unimportant for the classification. Importantly, the observed rate of change in accuracy was proportional to the importance of the information perturbed at each step as expected. This analysis allowed us to understand the relative significance of different regions within the input gait sequence for the classification performance of CNN models.

#### 4.2.2 PD gait event assignment using LRP

**Figure 10:**
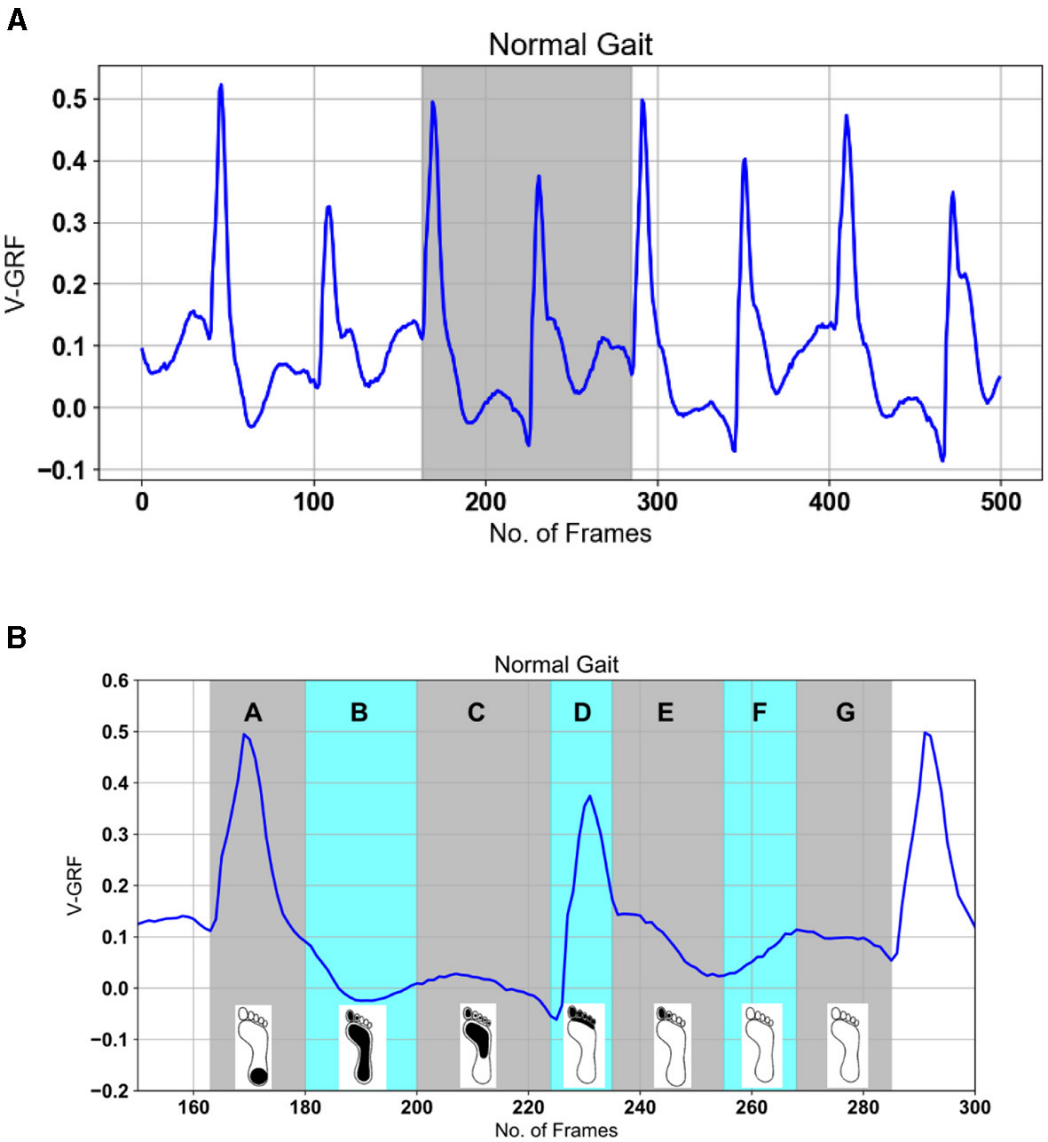
Gait events processed SA (see Equation 7) signal top. The highlighted gray area in **(A)** is explained in **(B)** based on gait events for one foot from Figure 4 as: A—heel strike, B—loading response or flat foot, C—mid-stance or single support, D—terminal stance or heel rising, E—pre-swing or double-limb support, F—initial swing and mid-swing or toe-off, G—terminal swing.

Gait GRF data take the form of periodic sequences, which are characterized as repetitive cycles for each foot. We note that the normal gait cycle is initiated by the heel strike of one foot, followed by other gait events described in Figure 1 and Table 1, in strict order. Therefore, the LRP-generated heat map of the temporal variations in the GRF signal can reveal which events in the gait cycle are most relevant for the classifications. Consequently, gait event assignment is best performed on the data sequences in Figure 10 after spatial averaging and standardization. A representative spatially averaged sensor signal sequence is shown in Figure 10A for a healthy subject. The highlighted gray area corresponds to one gait cycle, while the plotted signal is given by the spatial average (SA) metric, computed as follows:


(8) $$SA[n]=\frac{1}{18}\sum_{i=1}^{18}\left(x_{i}[n]\right)$$

Here, *x*_*i*_ are the readings from individual sensors, and *n* enumerates the frames in each sample. Recall that each foot has eight sensors attached (16 total) and the two sums one for each eight sensors for each foot are available giving 18 signals in total. Figure 10B shows the expanded gait cycle from Figure 10A with the gait events color-coded and labeled as per Figure 1 and Table 1.

##### 4.2.2.1 Interpretation

**Figure 11:**
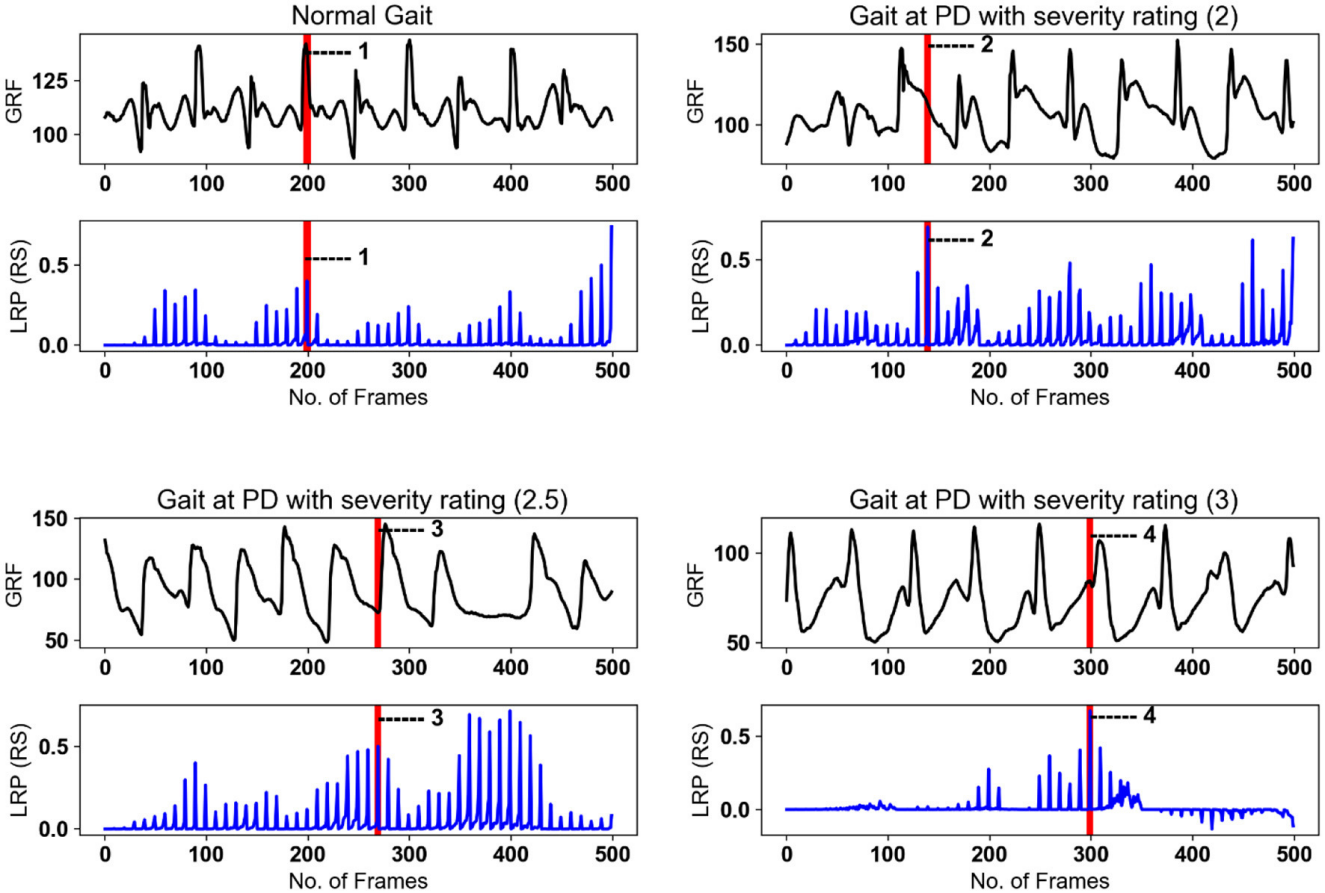
LRP method applied on randomly selected samples for healthy gait and three PD severity ratings. SA of gait spatiotemporal signals: black; SA for LRP relevance scores (RS) over the same temporal period: blue. Vertical red bars with number labels display consistency with gait events listed below with capital letters as per Figure 1 (Table 1) and Figure 10: 1—heal strike and foot flattening (A); 2—mid-stance and single support (C); 3—loading response after the double support interval (B), 4—terminal swing and ready for the heel strike (G).

The LRP scores highlight the regions of the input data that contribute significantly to the model's classification of PD severity stages. The plot of LRP scores in Figure 11 displays calculated *SA* (top panels) aligned against the relevant “LRP scores” *SA*, which consists of sharp peaks, well defined in the temporal domain, thus attributable to time-stamped gait events. Figure 11 displays the spatially averaged data signals for the four classes with their respective LRP score maps. The most prominent peaks are attributed to observable gait events, labeled in consistency with the gait cycle in Figure 1 and Table 1. It is observed that the model focuses on specific gait features related to severity, such as changes in stride length, gait speed, and variability, to make accurate predictions. These are further discussed in Section 5.

#### 4.2.3 Cognitive load impact on gait event assignment using LRP

The focus of this section is to identify the features picked up by the model to classify gait under cognitive load. To obtain accurate LRP relevance scores *Ri*, the true positive prediction of the model should be high. Therefore, the gait class with a high positive rate is considered for LPR analysis. The learned CNN model parameters in experiments 2 and 4 were frozen for LRP analysis. Experiment 3 is to check whether there is a variation in gait within a subject; therefore, it is not considered for LRP analysis. LRP sequential preset a flat (LRP-SPF) based on the **XAI Selection** criteria was utilized for this part as it has shown sensitivity to gait inconsistency. The iMagiMat system captures a sequence of periodic events as distinct, but similar cycles for each foot. This spatiotemporal sequence is generated by the change of light transmission intensity in the POF sensors: $x_{i}=\left[\begin{array}{c} x_{1}\&\ldots \&x_{116} \end{array}\right]\in {\mathbb{R}}^{n\times 116}$. However, a typical interpretation of the gait cycle, based on visual observation, is derived much less from the spatial component than the temporal one. Thus, to progress toward interpreting the CNN classifications in terms of observable gait events, we average over the spatial domain using Equation 8.

**Figure 12:**
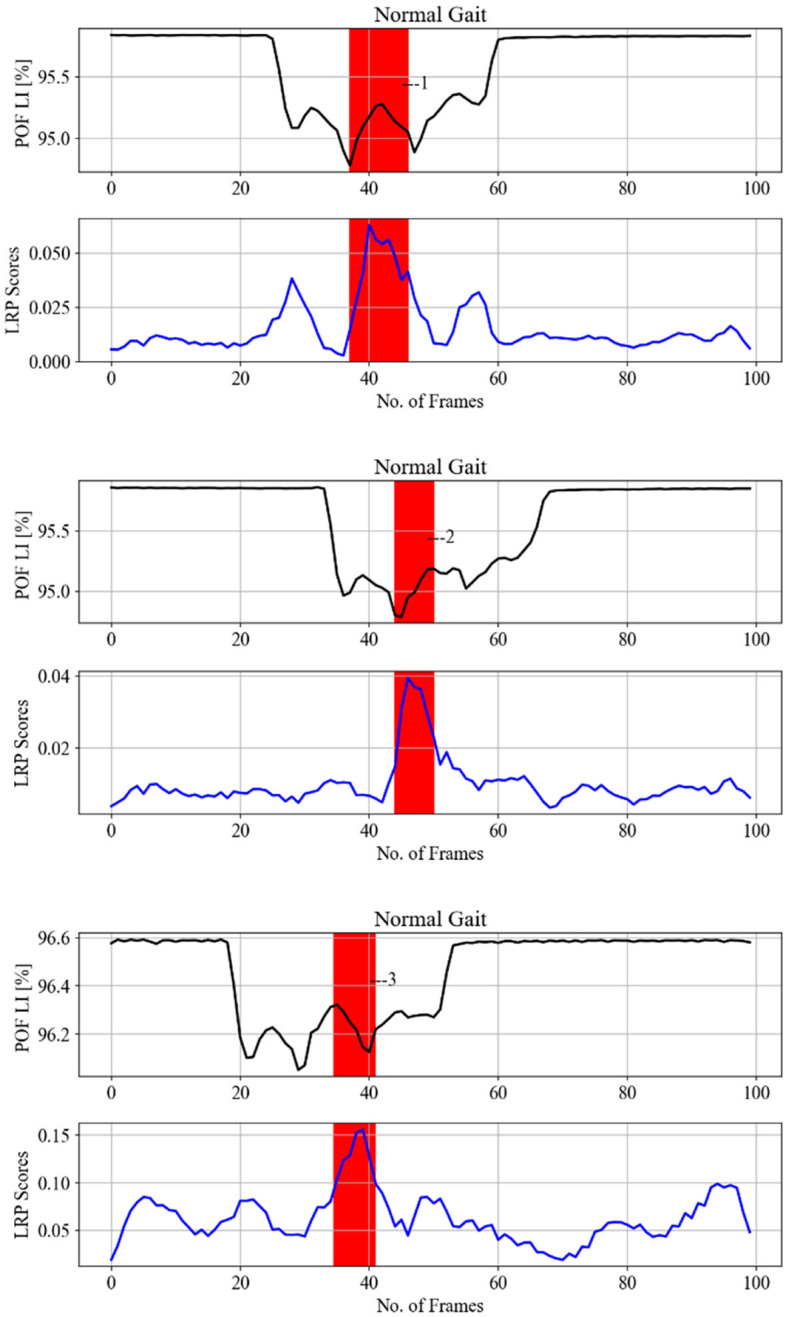
LRP methods applied on normal gait samples (from different subjects) from experiment 2 testing data, to identify gait events relevant to the CNN prediction to classify the cognitive load impact on gait. Gait events are 1,2,3—loading response or foot flat and double support.

**Figure 13:**
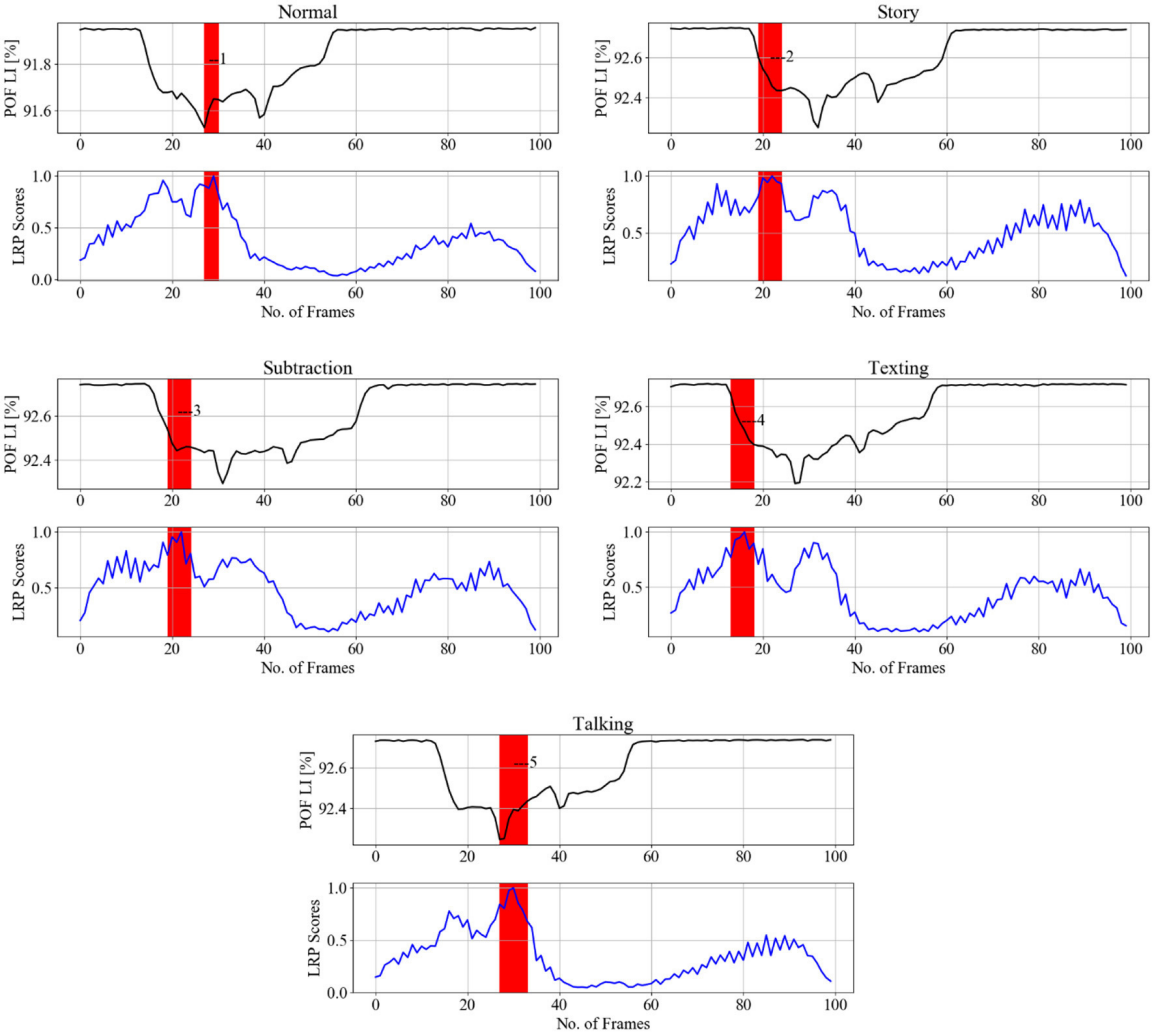
LRP methods applied on a single subject from experiment 3 testing data (each column is one pair), to identify gait events relevant for the CNN prediction to classify the cognitive load impact on gait. SA of gait spatiotemporal signals: black; SA for LRP relevance signals over gait temporal period: blue; POF LI (plastic optical fiber light intensity). Vertical red bars with numbers display correspondence to gait events as per Figure 15: 1, 5—loading response or foot flat and double support, 2, 3, 4—loading response or foot flat and single support.

**Figure 14:**
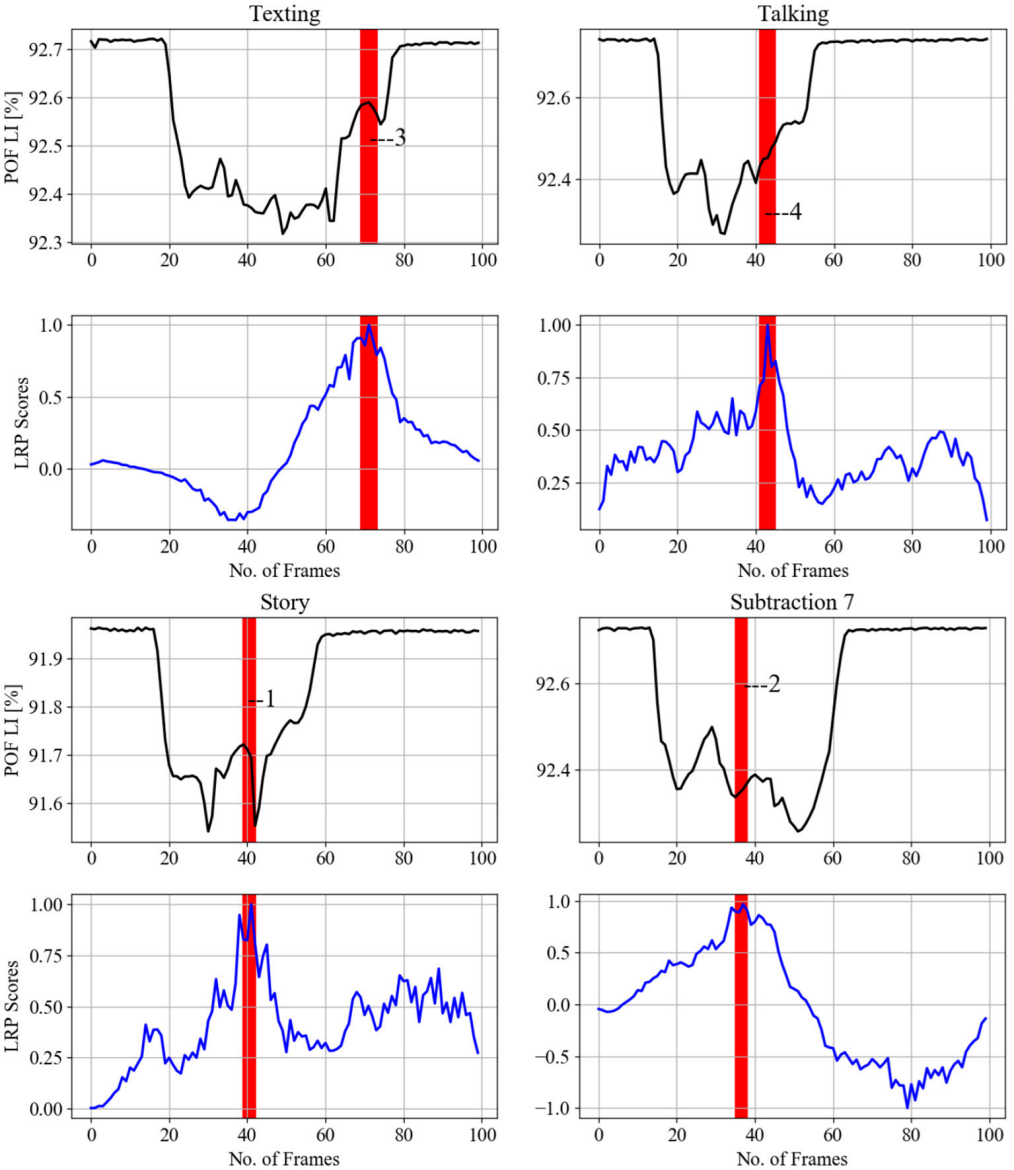
LRP methods applied on a single subject from experiment 4 testing data (each column is one pair), to identify gait events relevant for the CNN prediction to classify the cognitive load impact on gait. Gait events are as follows: 1—heel strike, 2—toe-off, 3—between foot swing and opposite heel strike, 4—between double support and toe-off.

Figure 12 displays randomly selected samples of normal gait classified with 100% true positives in experiment 2; Figure 13 shows predicted gait samples in experiment 3 for a single. Figure 14 displays randomly selected subjects for comparison of dual tasking with a normal gait. The top panels in Figures 12–14 display calculated *SA* aligned against the relevant “LRP scores” *SA*, generated from the calculated LRP scores and displayed in the bottom panels (to be discussed further in Section 5). The SA temporal sequences have different values on the *y-axis* due to the nature of the captured gait signal, which is influenced by the individual anthropometry of subjects.

##### 4.2.3.1 Interpretation of results

The LRP heatmaps demonstrate the regions in the input data that contribute significantly to the model's decision regarding cognitive load impact on gait. The model appears to focus on variations in gait features influenced by cognitive load, providing insights into the relationship between cognitive demand and gait characteristics.

The presented experiments demonstrate the effectiveness of the CNN model in various gait-related tasks, including PD identification, severity staging, subject identification, and assessing cognitive load impact on gait. The high F1-scores obtained in each experiment indicate the capability of model to make accurate predictions. The LRP analysis provides interpretability by highlighting important regions in the input data for decision-making. In the PD gait identification experiment, the model seems to focus on specific patterns in ground reaction forces related to PD-associated abnormalities. In PD severity staging, the model relies on gait features indicative of severity, such as stride length and variability. For subject cognitive load identification, the model captures unique gait patterns for each individual, and in assessing cognitive load impact, it considers variations influenced by cognitive demand. The ensemble approach consistently shows comparable or improved performance over the single model, indicating its effectiveness in enhancing predictive accuracy. The mean F1-scores across experiments suggest the model's robustness in handling diverse gait-related tasks. Overall, the presented CNN model, accompanied by LRP analysis, provides a powerful tool for gait analysis in the context of Parkinson's disease and related tasks. Further research and validation on larger datasets and diverse populations would contribute to the generalizability and applicability of the proposed model. Additionally, real-world deployment considerations, such as model interpretability in clinical settings, should be explored for practical implementation.

## 5 Discussion

The study presented delves into the promising realm of explainable artificial intelligence (AI) and deep learning methods for predicting gait deterioration. The focus is on identifying the impact of cognitive load and Parkinson's disease (PD) on gait patterns, and this is achieved by analyzing spatiotemporal data obtained from sensors placed under the feet. To carry out this investigation, convolutional neural networks (CNNs) were utilized. These powerful neural networks can effectively learn from complex spatiotemporal data and produce highly accurate predictions. In addition, the CNNs were perturbed to provide insights into the features within the spatiotemporal gait ground reaction force (GRF) signals that are most relevant to the predictions of the models. The results of this study are presented in detail in the following sections, with each data classification and perturbation analyzed and discussed in depth.

### 5.1 PD data

The spatiotemporal signal in Figures 4A, 10 implies that gait has normal events. Abnormal gait, otherwise difficult to detect visually, can be detected by machine learning, in alignment with the knowledge of the ground truth labels. However, the magnitude of GRF in Newton shows a decrease attributable to the severity of PD. The main objective of this study was to find the best deep learning model for PD severity rating and relate the model predictions to the gait cycle events shown in Figure 3.

Research toward machine learning classifications from PD data, specifically PhysioNet data, is based on the use of manual feature extraction methods with classical machine learning methods as shown in Table 8. The best classification results from manual extraction are reported in Abdulhay et al. (2018) using SVM classifier (92.7%). Our study on PD severity classification reported in Table 5 displays that the CNN outperformed the SGD, KNN, GPC algorithms, and LSTM. In this study, we explore three CNN architectures for automatic extraction and LRP analysis. The proposed CNNs identified PD, as well as rated the severity of the deviation from healthy gait, achieving better classification performance with an F1-score of 98% for each dataset and for the datasets combined with different random states (see Table 5). The best classification accuracy is achieved with the parallel CNN, with mean performance and standard errors of 95.5 and 0.28%, respectively. Additionally, the parallel CNN exhibit robustness at perturbation with Gaussian noise as shown in Figure 7. This suggests that the model is adequate for detecting gait deterioration from the spatiotemporal GRF signal. As an additional substantial enhancement, our LRP approach allows classification results to be related to visual observations similar to those established in medical practice to diagnose PD. In this section, we present key findings from our analysis, supported by visual representations. Figure 10 illustrates the spatiotemporal signal extracted from PD data, providing insights into the gait patterns of individuals with Parkinson's disease.

**Table 8:** PD classification results on PhysioNet three datasets.

**References**	**Methods**	**Accuracy (%)**
Abdulhay et al. (2018)	SVM	92.7
Jane et al. (2016)	Q-BTDNN	91.5
Ertugrul et al. (2016)	1D-LBP + MLP	88.89
Medeiros et al. (2016)	PCA	81.00
Wu et al. (2017)	SVM	84.48
This study	Parallel CNN	95.5 ± 0.28

SVM, support vector machine; 1D-LBP+MLP, shifted 1D-local binary patterns + multi-layer perceptron; PCA, principal component analysis; Q-BTDNN, Q-back-propagated time delay ANN.

Moving on to Figure 10B, we delve into the gait cycle events identified in PD data. These events play a crucial role in understanding the dynamics of gait abnormalities associated with Parkinson's disease.

To further refine our analysis, Figure 11 presents gait cycle events specifically categorized for PD severity staging. This categorization allows for a nuanced exploration of how gait characteristics vary across different stages of Parkinson's disease. These figures serve as visual aids to enhance the comprehension of our findings and contribute to the broader understanding of gait abnormalities in the context of Parkinson's disease.

**(1) PD Severity Level 0 (Healthy Gait):** The CNN classifies the raw spatiotemporal signals as healthy or within three severity ratings as shown in the confusion matrix (Figure 5). The best LRP method is selected by applying a perturbation technique, which detects the highest sensitivity to removal of information from the input data sequence (Figure 9). The selected LRP-SPF was found to be superior to well-known methods such as deconvolution and guided backpropagation.

Among the CNN architectures (Figure 3), the parallel CNN model shows the steepest decrease in the perturbation procedure. Therefore, that model is learned and used to generate the heatmap or relevance for randomly selected samples (Figure 11). The gait cycle events identified as key at each level of PD severity are listed below:

**PD Severity Level 0 (Healthy Gait):** (1) Heel strike and foot flattening (A).

This indicates that the healthy person's ability to maintain balance is stronger than the PD patients', with strong balance suggesting that the forces are applied rhythmically to achieve the lower limbs' synchronized movement with stable posture.

**PD Severity Level 2:** (1) Mid-stance and single support (C).

The heatmap shows that the subjects affected with PD level 2 have a weaker balance in single support, where this feature is marked by the model by 96% F1-score.

**PD Severity Level 2.5:** Loading response after the double-support interval (B).

This shows that the subject has weaker foot landing or flat foot landing after the balance is compromised by the single support.

**PD Severity Level 3:** (4) Terminal swing and ready for the heel strike (G).

Here the balance is compromised by weak GRF resulting from unstable body posture and implies a high risk of falling. This conclusion is based on linking the stages of PD in Wang et al. (2023) (description of how the stage of PD affects the body posture during gait using visual observation) to the events that are highlighted by the model for a certain PD severity.

**(2) Interpretation of Classifications:** The above markers for classification align with the observations in the literature that PD-induced gait GRF deterioration affects body balance and posture. The latter is with the closest relevance to gait events identified by the heat maps in Figure 11 as the highest LRP scores, while the other gait events are less significant to the classifications. It is worth mentioning that these markers are identical by 95.5% in 1,281 samples, such that the removal of these regions in the 95.5% of samples resulted in a strong decay in the model prediction. The interpretation given above is in very good agreement with the description of the Hoehn and Yahr Scale staging criteria as follows: ”Stage 0—No signs of disease, Stage 2—Symptoms on both sides but no impairment of balance, Stage 2.5—Mild symptoms on both sides, with recovery when the ‘pull' test is given (the doctor stands behind the person and asks them to maintain their balance when physically pulled backward), Stage 3—Balance impairment, mild-to-moderate disease, physically independent” (International Parkinson and movement disorder society, 2004). However, the staging criteria do not refer to the gait events adversely influencing the body's postural balance, due to the advancement of the disease.

### 5.2 iMagiMat data

#### 5.2.1 Classification of gait signatures under cognitive load

The present study investigates the importance of cognitive load influence on gait inconsistency. We present a comparison of classification performance between five types of gait: normal and under cognitive load in four different tasks. CNNs not only outperform, unsurprisingly, the classical classifier methods but also achieve an F1-score of 92% for normal gait (Figure 6 and Table 9) for cognitive load impact on gait in experiment 2 with 21 healthy adult data. Understandably the variation in the other cognitive demanding tasks gait is varying among subjects as each subject has a different way of dual taking.

**Table 9:** F1-score predictions for comparison of CNN with classical classifiers.

**Classifier**	**Experiment 3**	**Experiment 2**
SGD	77%	42%, *N* = 47%
KNN	87%	51%, *N* = 81%
GPC	5%	22%, *N* = 0%
CNN	100%	50%, *N* = 92%

N, True positive prediction of normal gait.

Experiment 3 is, in essence, an extra validation of the adequacy of the spatiotemporal sampling of GRF by the 116 sensors and their fusion as well as the classification performance of the trained models. An F1-score of 100% is achieved for most of the test data. Although Experiment 3 has the character of a sanity check, the results support the value of floor sensor gait data as a biometric. Experiment 2 is conducted to study the possibility of classifying cognitive load on healthy subjects. It has shown that normal gait is classified with a higher true positive rate compared to any of the classes of gait under cognitive load. This experiment also indicates that the achieved true positive rates in predicting normal gait are higher for the CNN model compared to the classical classifiers (see Figure 6 and Table 9). Samples obtained under cognitive load are hard to fit due to the inconsistency of gait pattern changes among the subjects.

The results from the first two experiments suggest that while the dual-task data obviously contributes to the high F1-scores in experiments 2 and 3, it results in substantially degraded true positive rates in experiment 2. However, experiment 3 shows that when classifications are within a single subject the performance is notably better: for 16 subjects (out of 21) the gait under cognitive load the F1-score ranges between 80 and 100%, with the remaining five subjects the range being between 69 and 77%.

These observations can be discussed in the light of humans having a natural gait pattern evolved over millions of years; however, changes in gait when experiencing cognitive load at any particular instance are specific to the individual, expressing their response to the impaired ability to process cognitive information (Chopra et al., 2018). In experiment 4, we use binary classifications (see Table 6) to distinguish normal gait from gait under the 4 variants of cognitive load. The best classification results are obtained when the model learns normal or dual-task gait features for a single subject. This implies that although learned gait features under cognitive load may not be readily portable across subjects, they are consistent for each individual and can contribute substantially for correct subject classifications; however, the accuracy drops if more subjects are involved.

**Figure 15:**
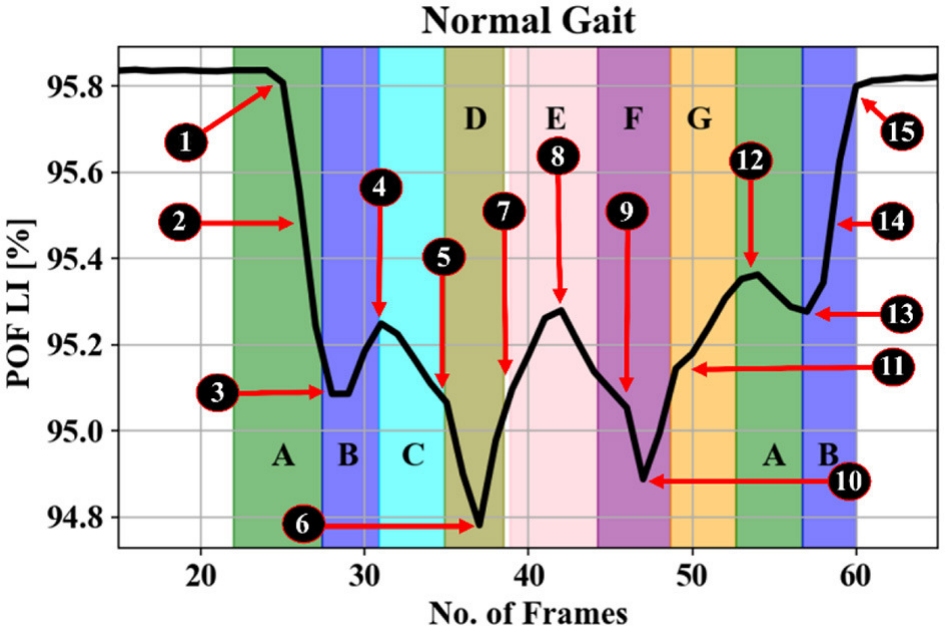
Representative gait cycle spatial average of spatiotemporal signals (see Equation 8). Gait events recorded by iMAGiMAT sensors in a typical full gait cycle of two steps (Figure 4: A, B, C, D, E, F, G): 1—heel strike, 2—foot-flattening, 3—single support, 4—opposite heel strike, 5—opposite foot-flattening, 6—double support, 7—toe-off, 8—foot swing, 9—heel strike, 10—double support, 11—toe-off, 12—foot swing, 13—opposite heel strike, 14—single support, 15—toe-off.

Figures 12–14 provide the link between the LRP relevance scores (“heat map”) and the time sequence of the calculated *SA* signal in a single gait cycle window. The LRP score maxima are suitable pointers to the parts of the gait cycle which are most relevant for the classifications. For accurate heat maps of a specific gait class the model's true positive prediction in the confusion matrix must be close to 100% for most of the testing samples, which points to the results from experiment 2 (Figure 12), for normal gait heat maps—in Figure 13 and experiment 3 for a single subject predicted gait under the 4 variants of cognitive load. Focusing just on one complete gait period (two steps) is justified by the fact that on multiple repetitive occasions each subject will initiate a gait cycle (see full description of the gait cycle Figure 1 and Table 1) by performing a heel strike, strictly followed by other gait events described in Figure 15 and ending in a toe off.

The indication of events numbered 1, 2, 3 on Figure 12 implies that normal gait identified by loading response or Foot flat and double support for 21 subjects. This gait event is marked by the model by 92% true positive (see Figure 12) to distinguish normal gait from 4 cognitive load classes. Figure 13 indicates that loading response has high relevance for assigning a gait signature to one out of the 21 subjects gait samples, notably even under cognitive load, as indicated by with gait events numbered from 1 to 5. The indicated gait events are 1,5—loading response or foot flat and double support, 2,3,4—loading response or foot flat and single support. Figure 12 displays the binary classification of randomly selected subject gait events as: 1—heel strike, 2—toe-off, 3—between foot swing and opposite Heel strike, 4—between double support and toe-off. Figure 12 shows cognitive load gait samples for one subject as per experiment 4 summarized as follows:

1. Gait while listening to story: Heel strike is significant for distinguishing listing to story from normal walking.
2. Gait while performing serial 7 subtraction: Toe-off is significant for distinguishing 7 subtraction from normal walking.
3. Gait while texting in smart phone: the transition from foot swing to opposite Heel strike is significant for distinguishing texting from normal walking.
4. Gait while talking: the transition from double support to Toe-off is important to distinguishing talking from normal walking.

Figure 13 indicates that loading response has high relevance for assigning a gait signature to one out of the 21 subjects gait samples, notably even under cognitive load, as indicated by with gait events numbered from 1 to 5. The indicated gait events are 1,5—loading response or foot flat and double support, 2,3,4—Loading response or foot flat and single support. Figure 12 displays the binary classification of randomly selected subject gait events as: 1—heel strike, 2—toe-off, 3—between foot swing and opposite heel strike, 4—between double support and toe-off.

Overall, the LRP analysis indicates that subjects' normal gait is characterized by loading response, while the other cognitive load gait classes are classified by landing or lifting the feet on/from the surface of the iMagiMat system. For subject dual tasking, there are many second relevant scores used to predict the cognitive load of the subject based on gait signature.

## 6 Conclusion

In conclusion, this study demonstrates the potential of explainable artificial intelligence (XAI) and deep learning methods in predicting gait deterioration. The use of convolutional neural networks (CNNs) on spatiotemporal data obtained from sensors under the feet proves to be effective in identifying the impact of cognitive load and Parkinson's disease (PD) on gait patterns. The proposed CNN architectures show robustness and achieve high classification accuracy for PD severity and cognitive load classification. The local relevance propagation (LRP) analysis provides valuable insights into the features of the spatiotemporal gait ground reaction force (GRF) signals that are most relevant to the model's predictions. The identified gait events and their relevance scores align with existing literature on PD-induced gait deterioration and cognitive load effects on gait. Additionally, the perturbation analysis validates the robustness of the model predictions, and the comparison of LRP methods highlights the effectiveness of the selected LRP-SPF method. The study contributes to the understanding of the relationship between gait events, PD severity, and cognitive load providing a foundation for further research in the field of gait analysis and neurodegenerative diseases. The findings suggest that the proposed model can not only classify gait patterns accurately but also reveal the specific features contributing to these classifications. The experiments conducted in this study shed light on the challenges associated with gait classification under cognitive load. Overfitting observed in the learning curve underscores the importance of addressing the variability in gait patterns induced by cognitive tasks across different subjects. Despite the challenges, the model exhibits promising performance, particularly in distinguishing normal gait from cognitive-loaded gait patterns. The binary classifications in Experiment 5 further emphasize the potential of the model for subject-specific gait analysis. The consistency of learned gait features within individuals suggests the applicability of the model for personalized gait assessments, although caution is warranted when generalizing across a larger population. The interpretation of classifications through LRP heatmaps reveals the relevance of specific gait events in distinguishing between normal and cognitive-loaded gaits. Loading response emerges as a critical gait event for identifying normal gait, while other events such as heel strike and toe-off play distinct roles in classifying cognitive-loaded gaits. The perturbation analysis validates the robustness of the model against the removal of relevant information. The ability of the model to maintain high performance in the presence of random perturbations suggests that it focuses on genuine gait features rather than noise. In conclusion, this comprehensive study not only demonstrates the effectiveness of deep learning models in gait analysis by achieving 98% classification results but also provides interpretability through LRP analysis using perturbation analysis to result in a robust model. The combination of accurate classification, subject-specific insights, and robustness to perturbations positions the proposed model as a valuable tool in clinical settings for assessing gait abnormalities associated with cognitive load and neurodegenerative diseases.

## Data Availability

The raw data supporting the conclusions of this article will be made available by the authors, without undue reservation.
